# Proportion and predictors of remission and recovery in first-episode psychosis: Systematic review and meta-analysis

**DOI:** 10.1192/j.eurpsy.2021.2246

**Published:** 2021-11-03

**Authors:** Ana Catalan, Anja Richter, Gonzalo Salazar de Pablo, Julio Vaquerizo-Serrano, Gonzalo Mancebo, Borja Pedruzo, Claudia Aymerich, Marco Solmi, Miguel Á. González-Torres, Patxi Gil, Philip McGuire, Paolo Fusar-Poli

**Affiliations:** 1 Mental Health Department, Biocruces Bizkaia Health Research Institute, Basurto University Hospital, Facultad de Medicina y Odontología, Campus de Leioa, University of the Basque Country, UPV/EHU, Barakaldo, Spain; 2 Department of Psychosis Studies, Institute of Psychiatry, Psychology and Neuroscience, King’s College London, London, United Kingdom; 3 Department of Child and Adolescent Psychiatry, Institute of Psychiatry and Mental Health, Hospital General Universitario Gregorio Marañón School of Medicine, Universidad Complutense, IiSGM, CIBERSAM, Madrid, Spain; 4 Early Psychosis: Interventions and Clinical-Detection (EPIC) Lab, Department of Psychosis Studies, Institute of Psychiatry, Psychology & Neuroscience, King’s College London, London, United Kingdom; 5 Department of Child and Adolescent Psychiatry, Institute of Psychiatry, Psychology and Neuroscience, King’s College London, London, United Kingdom; 6 Department of Psychiatry, Basurto University Hospital, Bilbao, Spain; 7 Department of Psychiatry, University of Ottawa, Ontario, Canada; 8 Department of Mental Health, The Ottawa Hospital, Ontario, Canada; 9 Ottawa Hospital Research Institute (OHRI), Clinical Epidemiology Program, University of Ottawa, Ottawa, Ontario; 10 Mental Health Department, Biocruces Bizkaia Health Research Institute, Early Intervention Service, Bizkaia Mental Health System, Bilbao, Spain; 11 OASIS Service, South London and Maudsley NHS Foundation Trust, London, United Kingdom; 12 Department of Brain and Behavioral Sciences, University of Pavia, Pavia, Italy; 13 National Institute for Health Research, Maudsley Biomedical Research Centre, South London and Maudsley NHS Foundation Trust, London, United Kingdom

**Keywords:** First-episode psychosis, predictors, psychosis, recovery, remission

## Abstract

**Background:**

To determine the proportion of patients in symptomatic remission and recovery following a first-episode of psychosis (FEP).

**Methods:**

A multistep literature search using the Web of Science database, Cochrane Central Register of Reviews, Ovid/PsychINFO, and trial registries from database inception to November 5, 2020, was performed. Cohort studies and randomized control trials (RCT) investigating the proportion of remission and recovery following a FEP were included. Two independent researchers searched, following PRISMA and MOOSE guidelines and using a PROSPERO protocol. We performed meta-analyses regarding the proportion of remission/recovery (symptomatic plus functional outcomes). Heterogeneity was measured employing *Q* statistics and *I*^2^ test. To identify potential predictors, meta-regression analyses were conducted, as well as qualitative reporting of studies included in a systematic review. Sensitivity analyses were performed regarding different times of follow-up and type of studies.

**Results:**

One hundred articles (82 cohorts and 18 RCTs) were included in the meta-analysis. The pooled proportion of symptomatic remission was 54% (95%CI [30, 49–58]) over a mean follow-up period of 43.57 months (SD = 51.82) in 76 studies. After excluding RCT from the sample, the proportion of remission remained similar (55%). The pooled proportion of recovery was 32% (95%CI [27–36]) over a mean follow-up period of 71.85 months (SD = 73.54) in 40 studies. After excluding RCT from the sample, the recovery proportion remained the same. No significant effect of any sociodemographic or clinical predictor was found.

**Conclusions:**

Half of the patients are in symptomatic remission around 4 years after the FEP, while about a third show recovery after 5.5 years.

## Introduction

After the first-episode of psychosis (FEP), the disorder can display a severe cognitive and behavioral decline that worsens symptoms and poor functioning [1,2]. Outcomes are highly influenced by early interventions’ timing and efficacy, which can improve symptoms and restore social and occupational functioning [3–5]. Accumulated evidence indicates that the early years after a FEP are critical for remission and recovery in psychosis [4,6]. Over the past decade, the criteria of The Remission Schizophrenia Working Group (RSWG) have been adopted as a common measure of symptomatic remission in clinical studies [7,9]. According to the RSWG, symptomatic remission is defined based on a fixed threshold for symptom severity (only mild or absent symptoms) and a time component (sustained for at least 6 months) [10].

While symptomatic remission can lead to recovery [10,11], the latter is more broadly conceived. Contrary to remission, recovery still lacks an accepted and validated definition [12]. There is consensus that symptomatic remission should be one of the components to be included in a definition of recovery [13]. Additionally, a consensus initiative suggested that improvement in independent living and in social and occupational/educational functioning for more than 1 year are core defining features of recovery [12].

Given the clinical relevance of symptomatic remission and recovery in FEP, considerable research has also been undertaken to identify their predictors. In a previous meta-analysis on FEP patients, 109 potential predictors of clinical outcomes were analyzed [14]. While this study concluded that medication nonadherence, longer duration of untreated psychosis (DUP), and substance misuse were core risk factors of relapse [14], no remission or recovery predictors were analyzed in FEP. Another meta-analysis [15] showed that cognitive deficits, concurrent remission and both positive and negative symptoms were predictors of recovery in FEP. However, in this meta-analysis, recovery was broadly defined by global or social functioning measures, for example, Global Assessment of Functioning (GAF), while changes in the severity of symptoms were not incorporated [15].

To our knowledge, four meta-analyses [15–18] and three systematic reviews have investigated symptomatic remission and recovery in FEP or schizophrenia [19–21]. Overall, these studies are limited by a variety of outcome definitions used [20], are heterogeneous with regard to individuals included in the analysis (first-episode or multi-episode) [17], included both prospective and retrospective studies and searched the literature only until 2016 [16].

The current systematic review and meta-analysis address the gaps mentioned above. We included prospective studies until 2020 with first-episode psychosis spectrum disorder participants with standardized definition of remission/recovery. The first aim of this study was to assess pooled proportion of symptomatic remission and recovery in FEP. The second aim was to identify potential predictors of remission and recovery in FEP.

## Methods

This study was conducted in accordance with Preferred Reporting Items for Systematic Reviews and Meta-analyses (PRISMA, Supplementary Table S1) [22] and Meta-analysis for Observational Studies in Epidemiology (MOOSE) guidelines (Supplementary Table S2) [23], following EQUATOR Reporting Guidelines [24]. The study protocol was registered on PROSPERO (CRD42020182080).

### Search strategy and selection criteria

A systematic literature search was performed by two independent researchers (A.C., A.R.) using the following search terms: “predictor*” “response” OR “outcome” OR “prognosis” OR “response” OR “remission” OR “recovery” AND “psychosis” OR “schizophrenia” OR “schizophreniform” OR “first-episode psychosis” OR “early psychosis” AND “cohort” OR “case-control” OR “RCT” OR “clinical trial.” A search was conducted in the Web of Science (which includes Web of Science Core Collection, BIOSIS Citation Index, KCI—Korean Journal Database, MEDLINE, Russian Science Citation Index, and SciELO Citation Index), the Cochrane Central Register of Controlled Trials, and Ovid/ PsychINFO databases, from inception until November 5, 2020. We further searched references from included studies and reviews that were screened during the literature search. Abstracts identified were then screened, and after excluding those that were not eligible, the full-text of the remaining articles were retrieved for further inspection against the inclusion and exclusion criteria.

We included: (a) prospective studies with a minimum follow-up of at least 6 months to detect remission or recovery. According to a recent meta-analysis [5], we operationalized remission in the current study as: (a) stability of symptoms and/or, (b) minimum symptom severity for at least 6 months according to the RSWG remission criteria [10], and (c) study-defined (see Supplementary Table S4 for details); and recovery as: (a) symptom stability/minimum severity plus improved social, educational or vocational attainment, (b) study-defined (for details see Supplementary Table S4), (c) FEP patients (including nonaffective psychosis see below), and (d) peer-reviewed original studies published in English. FEP was defined as having the first contact with healthcare services (both inpatient and outpatient settings), or less than 5 years of disorder [6] and operationalized criteria established by a clinical standard classification (Diagnostic and Statistical Manual [DSM], International Classification Disease [ICD] or Research Diagnostic Criteria [RDC] [25]). When studies were reporting overlapping samples for the meta-analyses, the study with the longest follow-up period was included. If this was unclear, studies with the largest sample were included.

### Data extraction

Three researchers (J.V.-S., G.M., and B.P.) independently extracted data from all included studies, and any discrepancies were resolved by consensus or by a third author (G.S.P.). The variables extracted included: first author and year of publication, country, setting, topic investigated, diagnostic classification method (DSM, ICD, and RDC), sample size, age, gender, length of study follow-up, DUP, study design, baseline presence of comorbidity, predictors of remission or recovery, the proportion of FEP patients exposed to antipsychotic treatment at baseline, the severity of baseline positive/negative/general psychotic symptoms (mean scores and SD), criteria used to define remission and recovery, quality assessment (see below). We extracted the primary outcomes, defined as the proportions of individuals with FEP who met the criteria for remission or recovery (at each follow-up time point: 6–<12 months; 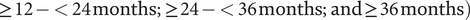 .

### Risk of bias (quality assessment)

Risk of bias was assessed using a modified version of the Newcastle–Ottawa Scale for case-control studies (Supplementary Table S3) and cohorts. Studies were awarded a maximum of eight points on items related to representativeness, sample size, group definition, validity, outcomes and representativeness, exposure, outcomes, follow-up period and loss to follow-up for cohort studies. The risk of bias assessment was conducted independently by two researchers (J.V.-S., G.S.P.). In cases of disagreement, a consensus was reached through discussion and, when not possible, to obtain consensus, a third researcher (A.C.) was included in the process.

### Strategy for data synthesis

Specific meta-analyses were: (a) proportion of remission and (b) recovery. Since heterogeneity was expected to be high, the random-effect model was employed [26]. Heterogeneity among studies was assessed using *Q* statistics, with the proportion of the total variability in effect size estimates evaluated using the *I*^2^ index (with an *I*^2^ > 50% representing significant heterogeneity) [27]. Publication biases were assessed for the proportion of remission or recovery by inspecting funnel plots and assessing Egger’s test [28]. Sensitivity analyses were performed to determinate the differences depending on whether the RSWG criteria were used to define remission or other broader definition was used; types of included studies (cohorts, case-control study, RCTs), and length of the follow-up period. In line with our hypotheses, we also performed (c) meta-regressions to estimate the association between potential predictors and remission and recovery proportions separately when data from at least 10 studies were available. The predictors included clinical variables (e.g., the severity of baseline positive/negative/general psychotic symptoms at baseline, DUP), sociodemographic variables (e.g., age and gender), percentage of FEP under antipsychotic treatment at baseline, length of follow-up, and NOS quality assessment. All analyses were conducted using STATA version 16 [29]. The significance level was set at a *p* < 0.05, two-sided.

We also provided a systematic narrative synthesis of the included studies around predictors of remission/recovery (supplementary results).

## Results

### Characteristics of the included studies

**Figure 1.**
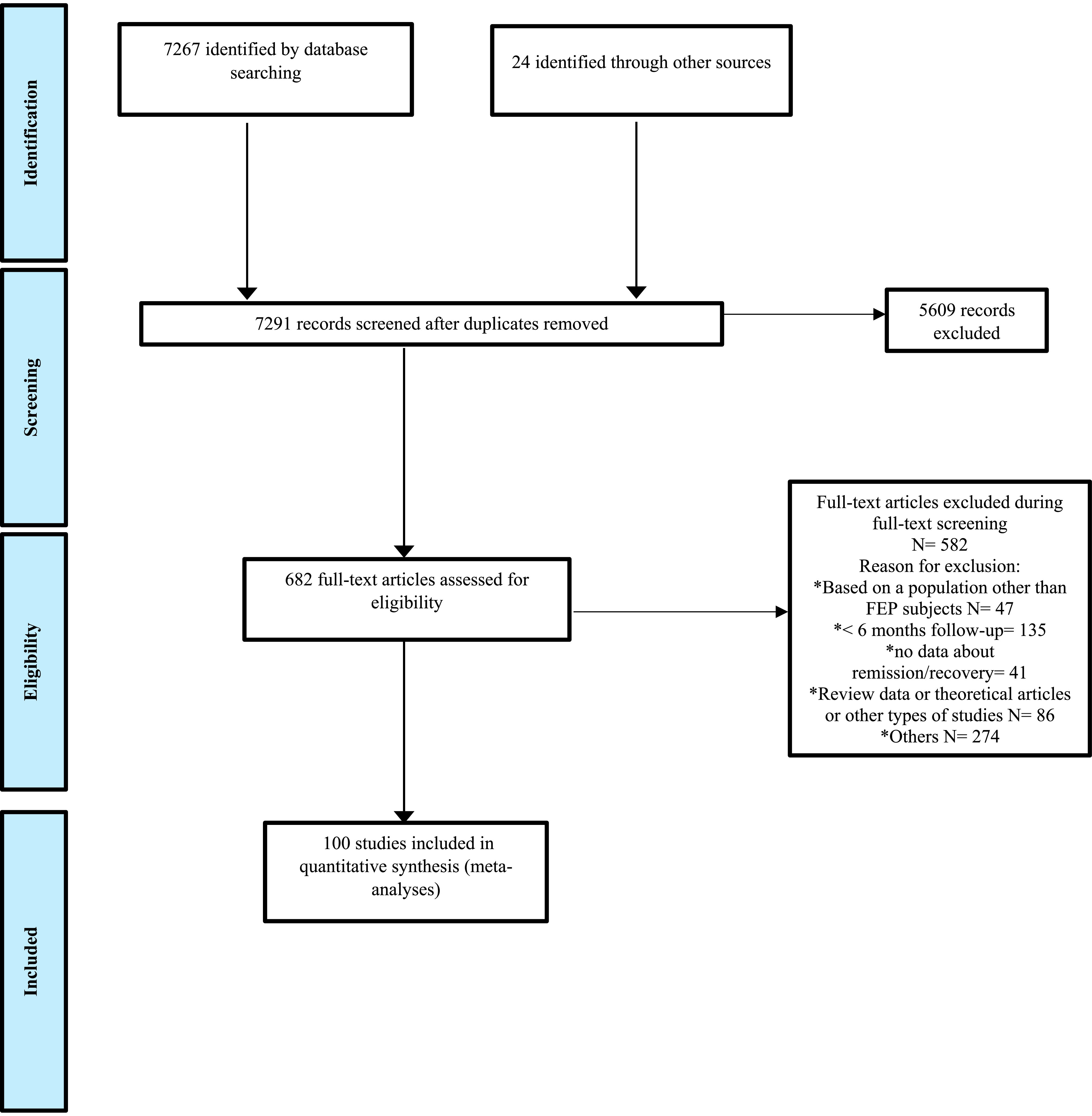
Preferred reporting items for systematic reviews and meta-analyses (PRISMA) flowchart outlining the study selection process.

Of 7267 articles identified, 100 (RCTs *k* = 18, observational studies *k* = 82) studies were included in the meta-analysis (*n* = 25375 FEP individuals; Figure 1). The studies’ sample size ranged from 16 to 2960; the mean age of included participants was 26.18 (SD = 3.93) years, and 67.8% of the sample was male in no overlapping samples. The characteristics of the included studies are detailed in Supplementary Tables S5 and S6.

Details of the operationalization criteria for remission and recovery are listed in Supplementary Tables S4 and S5. Most studies (*N* = 43, 57.33%) employed the RSWG criteria [10] to define remission: having mild or absent symptoms (symptom severity criteria) for at least 6 months (duration criteria). The remaining studies (42.66%) employed broader operational remission criteria to define significant symptomatic remission (24.14% of the studies using more than one instrument, see Supplementary Table S5) with no duration criteria.

Recovery was operationalized as symptomatic remission (16 studies [39.02%] using the RSWG criteria, and 24 studies [60.97%] using broader remission criteria as described above) and significant improvement in functioning. Functional improvement involved the following domains: functioning status (19 studies, 46.34%), living independently (13 studies, 31.71%), working or studying (21 studies, 51.23%), and social interactions (9 studies, 21.95%).

Forty-two studies described predictors of remission (Supplementary Table S7) and 28 studies described predictors of recovery (Supplementary Table S8). The most studied predictors were sociodemographic and clinical variables.

### Meta-analyses

#### Remission

We found 76 studies reporting on remission (RCTs *k* = 15, observational studies *k* = 59) (N FEP = 18528, Supplementary Table S5). The total remission proportion was 54% (95%CI [30, 49–58]; *I*^2^: 97.86%, *z* = 21.63; Supplementary Figure S1) with a mean follow-up of 43.57 months (SD = 51.82; range = 6–240). Significant heterogeneity was found between studies (Supplementary Figure S3), but no significant publication bias (Egger’s test *p* = 0.79).

Forty-two studies described predictors of remission (Supplementary Table S7). A systematic narrative synthesis of these studies is provided in supplementary results.

#### Recovery

We analyzed 40 articles with data reporting on recovery (RCTs *k* = 5, observational studies *k* = 35) (N FEP = 15064, Supplementary Table S6). A detailed description of the recovery definition used in these studies is shown in Supplementary Table S6. The total recovery proportion was 32% (95%CI [27–36]; *I*^2^ = 97.31%, *z* = 13.17; Supplementary Figure S2) with a mean follow-up of 71.85 months (SD = 73.54; range = 6–300). Publication bias for small studies was found (Egger’s test *p* = 0.02; Supplementary Figure S4).

Twenty-eight studies described predictors of recovery (Supplementary Table S8). A systematic narrative synthesis of these studies is provided in supplementary results.

#### Sensitivity analyses

**Figure 2.**
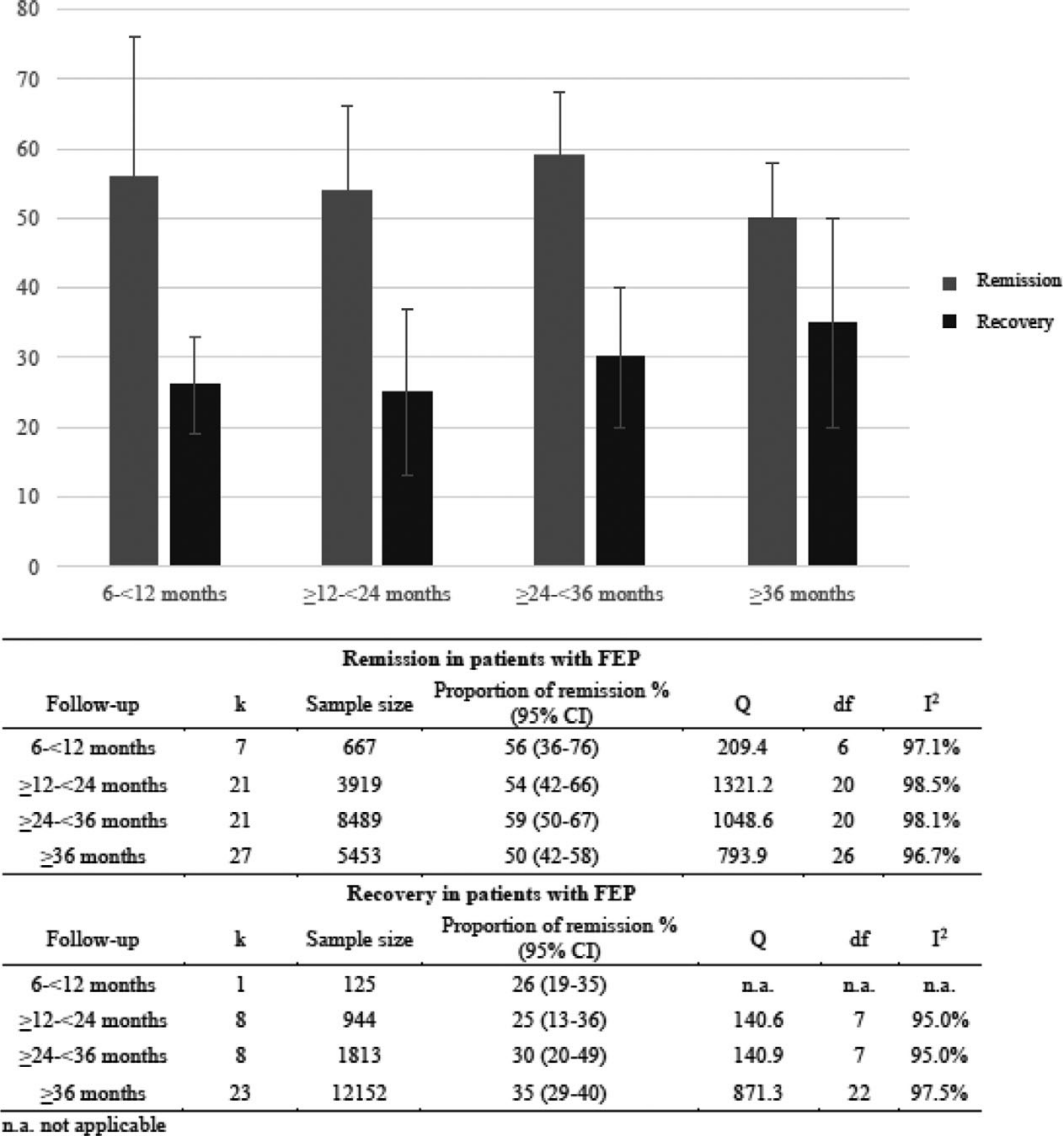
Meta-analytic symptomatic remission and recovery proportion in first-episode of psychosis (FEP) over time.

Sensitivity analyses were performed considering the duration of the follow-up period (Figure 2), the definition of remission (RSWG criteria [10] versus broader remission criteria), and the type of study (cohorts, case-controls, and RCT).

Sensitivity analysis on follow-up period showed that the remission proportion was slightly reduced with the longer duration of the follow-up (Figure 2). Depending on which remission criteria were applied, the proportion of remission varies (RWSG remission criteria: *k* = 39; 52% (95%CI [30,46–58]); broader remission criteria with no duration criteria: *k* = 37; 56% (95%CI [30,49–62]). After excluding 15 RCTs from the analysis, the remission proportion was 55%, 95%CI [30, 50–60] (*I*^2^: 97.93%, *z* = 19.58).

Regarding recovery, sensitivity analysis on follow-up period showed that the recovery proportion slightly increased the longer the duration of the follow-up (Figure 2). A sensitivity analysis of studies reporting recovery after 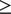 2 years and recovery at <2 years were performed [30]. Six studies assessed recovery status after more than 2 years (38%; 95%CI [30, 19–56]), while 34 studies assessed recovery proportion in less than 2 years (30%; 95%CI [26–35]). After exclusion of RCT, the recovery proportion was similar to the pooled proportion (32%, 95%CI [27–37]).

### Meta-regressions

The effect of different predictors of remission proportion is shown in Table 1. None of the predictors studied had a significant effect on remission. The meta-regression analyses showed that the proportion of patients in recovery was moderated by male sex (*ß* = 0.006; 95%CI [0.0003,0.0117], *p* = 0.04, *R*^2^ = 10.71) and by positive symptoms (*ß* = 0.0126; 95%CI [0.0041,0.0207], *p* = 0.002, *R*^2^ = 57.22; Table 2). These associations did not survive multiple comparison corrections (*p* < 0.002).

**Table 1.** Meta-regressions, effect of predictors on remission.

Name of predictor	*N* studies	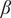	95% CI	*p*	*R*^2^
Follow-up (months)	76	−0.0004	[−0.0013, 0.0004]	0.270	0.24
NOS quality assessment	76	0.0179	[−0.0191, –0.0549]	0.343	<0.01
DUP median months	19	0.0004	[−0.0023, 0.0031]	0.767	<0.01
DUP mean months	29	−0.0031	[−0.0077, 0.0015]	0.189	2.81
Schizophrenia-spectrum diagnosis %	61	0.0004	[−0.0022, 0.0030]	0.763	<0.01
Sex male %	59	0.0013	[−0.0023, 0.005]	0.475	<0.01
Age	72	−0.0029	[−0.0138, 0.0079]	0.601	<0.01
Ethnicity Caucasian %	18	−0.0018	[−0.0058, 0.0022]	0.373	<0.01
Baseline positive symptoms PANSS	28	−0.0025	[−0.0155, 0.0105]	0.708	<0.01
Baseline negative symptoms PANSS	28	0.0042	[−0.0139, 0.0224]	0.645	<0.01
Functioning GAF	28	−0.0051	[−0.0119, 0.0016]	0.139	4.80
AP treatment baseline %	24	−0.0005	[−0.0027, –0.0018]	0.684	<0.01

*Note: p* < 0.002, after correction for multiple comparisons.

Abbreviations: DUP, duration of untreated psychosis; NOS, Newcastle–Ottawa Scale.

**Table 2.** Meta-regressions, effect of predictors on recovery.

Name of predictor	*N* studies	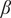	95% CI	*p*	*R*^2^
Follow-up (months)	40	0.0004	[−0.0004, 0.0012]	0.297	0.51
NOS quality assessment	40	−0.0196	[−0.0802, 0.0410]	0.527	0.00
DUP median weeks	11	−0.001	[−0.0032, 0.0032]	0.368	0.00
DUP mean weeks	12	−0.0022	[−0.0101, 0.0057]	0.59	0.00
Schizophrenia-spectrum diagnosis %	21	−0.0025	[−0.0058, 0.0007]	0.130	7.75
Sex male %	31	0.006	[−0.0003, 0.0117]	0.040	10.71
Age	36	−0.0023	[−0.0173, 0.018]	0.768	0.00
Baseline positive symptoms PANSS	11	0.0126	[0.0041, 0.0207]	0.002	57.22
Baseline negative symptoms PANSS	12	−0.0037	[−0.0194, 0.012]	0.644	0.00
Functioning GAF	17	0.0014	[−0.0109, 0.0139]	0.817	0.00

*Note: p* < 0.002, after correction for multiple comparisons.

Abbreviations: DUP, duration of untreated psychosis; NOS, Newcastle–Ottawa Scale.

### Quality assessment

The quality rating of the studies ranged from 2 to 7 (mean = 4.95; median = 5) on a modified version of the Newcastle–Ottawa scale (Supplementary Table S3).

## Discussion

To our knowledge, this is the largest systematic review of studies of remission and recovery in psychosis conducted to date. We found that more than half of the patients (54%) achieved remission 4 years after the first-episode, while around a third were in recovery after 5.5 years (32%). The most studied predictors of remission and recovery were clinical and sociodemographic variables.

Our results are consistent with those of a previous meta-analysis [16], although the proportion of remission and recovery is nominally lower (54 vs. 58% for remission and 32 vs. 38% for recovery). Another systematic review in FEP showed “good” outcomes for 42% of patients with psychosis and 31% of patients with schizophrenia [20], while a more recent systematic review of remission identified a remission proportion of 40% (range 17–78%) in FEP patients [19].

The current study has several advantages compared to previous reviews: the inclusion of RCT studies, inclusion of only prospective designs, and all studies reporting measures of recovery were classified. Additionally, we included more studies using the RSWG remission criteria (*N* = 43 compared to *N* = 25) [16].

Without antipsychotic treatments, most FEP cases display frequent poor outcomes [4,31]. However, with antipsychotic treatment, one study showed that remission was achieved by 70% of FEP patients after 6 months [13], while another study showed that 74% were in remission after approximately 9 months [32]. This meta-analysis highlights that the remission proportion could be lower than previously reported.

As we expected, the remission proportion decreased over time (56–50%, Figure 2), as relapse after a FEP is common [33], while the recovery proportion increased (26–35%). This could partially be explained by the fact that recovery involved other social and functioning goals, which require more time to be achieved [34]. Although a high proportion of patients can achieve remission in the short-term [16], only a minority of them get the full recovery in the long-term [35].

The most recent systematic review and meta-analysis of recovery showed a pooled proportion of 38% in FEP patients [16], and an earlier systematic review and meta-analysis revealed that only 13.5% of patients with schizophrenia met the criteria for recovery [17]. While the most recent systematic review and meta-analysis completed the literature search in 2016, yielding 3021 studies [16], the current study provides a more comprehensive, up to date overview of the literature on remission and recovery (current search yielded 7267 studies).

After excluding RCTs from the analyses, we showed that the remission and recovery proportion rates remained the same. This is surprising given the sample biases generally attributed to RCTs [36] (e.g., RCTs usually include adherent participants with no drug abuse or suicidal ideation). Observational studies could be overrepresented in the meta-analysis. Besides, the cohort studies also used active treatments, including early intervention programs [37–39].

While we did not find any predictors associated with remission, two were associated with recovery, namely male sex and positive symptoms. Previous studies did not find gender differences in the prognosis of psychosis after a FEP [40,41]. The association between higher scores of positive symptoms and increased recovery proportion may be related to the intrinsic heterogeneity of FEP cohorts. However, it is important to note that both the association with male sex and positive symptoms did not survive multiple comparison corrections and need to be investigated in future studies to draw meaningful clinical conclusions. Different negative psychotic symptoms trajectories in FEP patients have been described [42]. Negative psychotic symptoms are generally linked to worse outcomes in FEP [43]. However, only a small proportion of FEP subjects present a high level of persistent negative symptoms [44]; therefore, this effect could be underestimated in large samples of FEP.

Although there is extensive literature [45–48] reporting that DUP is related to a worse prognosis in patients with psychosis, we did not find such an association. Several studies suggest that the relationship between DUP and prognosis is based on other factors [49,50], such as positive symptoms. In line with our findings, a recent systematic review investigating remission/recovery proportion also did not find a relationship between DUP and better outcomes in psychosis [16], which could also be related to some inaccuracy in defining or measuring DUP. However, a recent umbrella review described suggestive evidence for a relationship between longer DUP and lower chance of remission and poorer functioning [51], but this result could be affected by lead-time bias, and thus, DUP would not be an important predictor of the outcomes and it could be more an indicator of illness stage [52].

The proportion of recovery in FEP is dramatically low. As an extended concept of remission, recovery has attracted interest as a desirable outcome in psychosis. It usually comprises symptomatic remission as a basic condition and functional improvements in vocational perspectives, independent living and social functioning [34,53]. Thus, recovery is a more complex concept than remission [30,54]. Unlike symptomatic remission, recovery encompasses multiple aspects of the patient’s life, including functioning and making it difficult to settle on a standard definition and develop reliable assessment criteria. Some clinicians think recovery can only be achieved when symptoms are mild or absent and remain that way for a prolonged time to not interfere with normal functioning in social activities and relationships [55]. Additionally, social and family circumstances, opportunities, and lifetime events contribute to extending the list of environmental factors that may influence recovery beyond clinical manifestations of schizophrenia [56,57]. The lack of a clear definition and assessment tools prevents from drawing strong conclusions regarding the feasibility of a therapeutic model based on the concept of recovery. However, empirical evidence on various therapeutic interventions suggests that many patients with schizophrenia can achieve recovery goals such as independent living and competitive employment and education in routine community settings [58]. Symptomatic recovery and functional recovery are different aspects that should be clearly differentiated to facilitate comparison between studies. Symptom and functional illness trajectories are independent, with some studies reporting lower functional recovery rates than symptomatic recovery rates [59]. This might suggest that these outcomes are likely to be influenced by different predictor variables and thus needing to be treated by different interventions. Researchers have combined commonly used scales such as the Global Assessment Functioning (GAF) scale and other objective indicators of lifetime achievements [60,61]. Despite the lack of a well-defined concept of recovery, some authors identified a trend toward a common archetype of the definition and factors associated with recovery and its applicability in clinical practice and clinical research [62].

In most cases, symptomatic remission is a prerequisite for recovery [63–66], so it might not be surprising that the recovery proportion is lower than those for remission [10]. Although the RSWG criteria to operationalize remission are widely used [9, 67–70], some studies used criteria for symptoms severity without applying a duration criterion [71], while other studies have used other outcome measures (e.g., CGI-S) [72].

### Limitations and strengths

This study includes a large sample of FEP and analyzed the proportion of remission and recovery. The main limitation of this study is the lack of operationalized definitions for recovery. Remission was operationalized in 2005 [10], and the majority of included studies used the RSWG criteria. However, there are other studies that applied broader remission criteria [63,73,74]. The definition of recovery is even more complicated. Some of the studies we examined used definitions that included functional remission [64,65] while others used much more exigent definitions, including having work and a relationship with a peer [14]. Some authors did not report the proportion of drop-out, the treatment used, premorbid adjustment, age of psychosis onset, or comorbidity in their samples which limited our set of different predictors being analyzed. In most studies, there was no information on the proportion of patients who met the remission or recovery criteria during the whole follow-up, limiting the final remission/recovery proportion. Moreover, psychosis is a long-term chronic disorder with negative outcomes frequently evolving over the whole lifetime [17]. A minor proportion of studies included a follow-up after 10 years which could impact remission/recovery rates and overestimate these rates for a subgroup of patients. Therefore, we conducted subgroup and meta-regression analyses to explore potential sources of heterogeneity. We showed that the main findings largely remained the same.

## Conclusions

This is the most up to date meta-analysis of remission and recovery proportion, and predictors of both outcomes in people with FEP. We showed that around half of patients with FEP reached symptomatic remission after 4 years, and about a third were in recovery after 5.5 years. Remission and recovery rates in FEP subjects remain low, indicating that more than half of patients do not achieve remission, and two-thirds do not achieve recovery at long-term follow-up. The early intervention services should implement strategies to improve long-term outcomes and detect patients who can benefit more from intensive care.

## Data Availability

The data that support the findings of this study are available from the corresponding author, A.C., upon request.

## Supplementary Materials

To view supplementary material for this article, please visit http://dx.doi.org/10.1192/j.eurpsy.2021.2246. click here to view supplementary material
