# Supplementary material for: Proportion and predictors of remission and recovery in first-episode psychosis: Systematic review and meta-analysis
Source: Eur Psychiatry. 2021 Nov 3;64(1):e69. doi: 10.1192/j.eurpsy.2021.2246 (PMC8668449; doi:10.1192/j.eurpsy.2021.2246)
Supplement: Supplementary file 1 [file S092493382102246xsup001.docx]

**Supplementary material**

[**Table S1**. PRISMA statement and checklist. 2](#_Toc65329203)

[**Table S2**. Moose checklist. 4](#_Toc65329204)

[**Table S3**. Risk of bias (quality) assessment using modified Newcastle Ottawa Scale for cross-sectional and cohort studies. 7](#_Toc65329205)

[**Results S1.** Systematic review 8](#_Toc65329206)

[**Table S4.** Remission/recovery criteria 10](#_Toc65329207)

[**Table S6.** Description of studies with recovery data. 16](#_Toc65329208)

[**Table S7.** Predictors of studies with remission data 21](#_Toc65329209)

[**Table S8**. Predictors of studies with recovery data. 28](#_Toc65329210)

[**Figure S1**. Forest plot remission data 34](#_Toc65329211)

[**Figure S2.** Forest plot recovery data 35](#_Toc65329212)

[**Figure S3**. Funnel plot for remission studies 36](#_Toc65329213)

[**Figure S4.** Funnel plot for recovery studies 37](#_Toc65329214)

This supplementary material has been provided by the authors to give readers additional information about their work.

**Table S1**. PRISMA statement and checklist.

| **Section/topic** | **#** | **Checklist item** | **Page** |
| --- | --- | --- | --- |
| **TITLE** | | |  |
| Title | 1 | Identify the report as a systematic review, meta-analysis, or both. | Title |
| **ABSTRACT** | | |  |
| Structured summary | 2 | Provide a structured summary including. as applicable: background; objectives; data sources; study eligibility criteria, participants, and interventions; study appraisal and synthesis methods; results; limitations; conclusions and implications of key findings; systematic review registration number. | Abstract |
| **INTRODUCTION** | | |  |
| Rationale | 3 | Describe the rationale for the review in the context of what is already known. | Introduction |
| Objectives | 4 | Provide an explicit statement of questions being addressed with reference to participants, interventions, comparisons, outcomes, and study design (PICOS). | Introduction |
| **METHODS** | | |  |
| Protocol and registration | 5 | Indicate if a review protocol exists, if and where it can be accessed (e.g. Web address), and, if available, provide registration information including registration number. | Methods |
| Eligibility criteria | 6 | Specify study characteristics (e.g. PICOS length of follow-up) and report characteristics (e.g. years considered. language. publication status) used as criteria for eligibility, giving rationale. | Methods |
| Information sources | 7 | Describe all information sources (e.g. databases with dates of coverage. contact with study authors to identify additional studies) in the search and date last searched. | Methods |
| Search | 8 | Present full electronic search strategy for at least one database, including any limits used, such that it could be repeated. | Methods |
| Study selection | 9 | State the process for selecting studies (i.e. screening. eligibility. included in systematic review, and, if applicable, included in the meta-analysis). | Methods |
| Data collection process | 10 | Describe method of data extraction from reports (e.g. piloted forms. independently. in duplicate) and any processes for obtaining and confirming data from investigators. | Methods |
| Data items | 11 | List and define all variables for which data were sought (e.g. PICOS funding sources) and any assumptions and simplifications made. | Methods |
| Risk of bias in individual studies | 12 | Describe methods used for assessing risk of bias of individual studies (including specification of whether this was done at the study or outcome level), and how this information is to be used in any data synthesis. | Methods |
| Summary measures | 13 | State the principal summary measures. | Methods |
| Risk of bias across studies | 15 | Specify any assessment of risk of bias (i.e. Newcastle-Ottawa Scale (NOS)). that may affect the cumulative evidence. | Methods |
| Additional analyses | 16 | Describe methods of additional analyses (e.g. sensitivity or subgroup analyses. meta-regression), if done, indicating which were pre-specified. | Methods |
| **RESULTS** |  |  |  |
| Study selection | 17 | Give numbers of studies screened, assessed for eligibility, and included in the review with reasons for exclusions at each stage, ideally with a flow diagram. | Results |
| Study characteristics | 18 | For each study, present characteristics for which data were extracted (e.g. study size. PICOS follow-up period) and provide the citations. | Results |
| Risk of bias within studies | 19 | Present data on risk of bias of each study and, if available, any outcome level assessment (see item 12). | Results |
| Results of individual studies | 20 | For all outcomes considered (benefits or harms), present for each study a summary data for each intervention group | Results |
| Synthesis of results | 21 | Present results of study analysed. | Results |
| Risk of bias across studies | 22 | Present results of any assessment of risk of bias across studies (see Item 15). | Results |
| Additional analysis | 23 | Give results of additional analyses, if done (e.g. sensitivity or subgroup analyses, meta-regression [see Item 16]). | Results |
| **DISCUSSION** |  |  |  |
| Summary of evidence | 24 | Summarize the main findings including the strength of evidence for each main outcome; consider their relevance to key groups (e.g. healthcare providers, users, and policy makers). | Discussion |
| Limitations | 25 | Discuss limitations at study and outcome level (e.g. risk of bias), and at review-level (e.g. incomplete retrieval of identified research. reporting bias). | Discussion |
| Conclusions | 26 | Provide a general interpretation of the results in the context of other evidence, and implications for future research. | Discussion |
| **FUNDING** |  |  | Discussion |
| Funding | 27 | Describe sources of funding for the systematic review and other support (e.g. supply of data), role of funders for the systematic review. | Discussion |

**Table S2**. Moose checklist.

| **Criteria** | | **Brief description of how the criteria were handled in the meta-analysis** |
| --- | --- | --- |
| **Reporting of background should include** | |  |
| √ | Problem definition | To examine at a meta-analytical level the remission and recovery rates after a First Episode-Psychosis (FEP).  To identify possible predictors of recovery and remission that specifically influence on these parameters. |
| √ | Hypothesis statement | We hypothesized that FEP group would have a significant remission rates and lower recovery rates. |
| √ | Description of study outcomes | Remission was operationalized in terms of symptomatic and/or functional improvement. Following the well-established Remission in Schizophrenia Working Group (RSWG) criteria[1], remission was defined as patients having mild or absent symptom severity and having mild or absent symptoms for at least 6 months. Besides, operational criteria proposed by authors from determinate cut-offs in the percentage of reduction of baseline scores on any validated psychopathology rating scale were also be included  Recovery has been operationalised in terms of symptomatic and functional improvement in social, occupational and educational domains. The definition of recovery included the symptomatic remission plus any kind of social and functional outcomes. The functional domains included (functional independence or personal autonomy, social relationships, being productive or professional activity, having a sense of empowerment, and environmental factors). Definitions offered by authors based on operationalised criteria were also included.  Predictors of remission and recovery were also extracted. |
| √ | Type of exposure or intervention used | We included individual studies that reported remission/recovery data in FEP population. |
| √ | Type of study designs used | Case-control studies, cohort studies, RCT, which investigate the remission and recovery rates in FEP. |
| √ | Study population | FEP population |
| **Reporting of search strategy should include** | |  |
| √ | Qualifications of researchers | The credentials of the investigators are indicated in the author list and in the acknowledgements. |
| √ | Search strategy, including time period included in the synthesis and keywords | We performed a multi-step literature search using the following keywords: "“predictor*” “response” OR “outcome” OR “prognosis” OR “response” OR “remission” OR “recovery” AND “psychosis” OR “schizophrenia” OR “schizophreniform” OR “first-episode psychosis” OR “early psychosis” AND “cohort” OR “case-control” OR “RCT” OR “clinical trial” from inception until 5th November 2020. |
| √ | Databases and registries searched | Web of Science database (Clarivate Analytics): Web of Science Core Collection. BIOSIS Citation Index. KCI-Korean Journal Database. MEDLINE. Russian Science Citation Index, PubMed and SciELO Citation Index. |
| √ | Use of hand searching | We hand-searched bibliographies of retrieved papers for additional references. |
| √ | List of citations located and those excluded, including justifications | Details of the literature search process are outlined in the results section and in the PRISMA flowchart. |
| √ | Method of addressing articles published in languages other than English | Only articles in English language were selected. |
| √ | Method of handling abstracts and unpublished studies | Original individual studies were included. Conference proceedings, reviews, editorials, clinical cases and unpublished studies were excluded. |
| √ | Description of any contact with authors | We did not contact corresponding authors to request additional data for this study. |
| **Reporting of methods should include** | |  |
| √ | Description of relevance or appropriateness of studies assembled for assessing the hypothesis to be tested | Detailed inclusion and exclusion criteria were described in the methods section. |
| √ | Rationale for the selection and coding of data | Data extracted from each of the studies were relevant to the population characteristics, study design, comparison group, exposure and outcomes. |
| √ | Assessment of confounding factors | Confounding factors were systematically assessed as stated in the methods of the study. |
| √ | Assessment of study quality. | We adapted the Newcastle-Ottawa Scale for the evaluation of cross-sectional and cohort studies. |
| √ | Assessment of heterogeneity | Heterogeneity was assessed with the I^2^ index. |
| √ | Description of statistical methods in sufficient detail to be replicated | Statistical methods are described in detail in the methods section. |
| √ | Provision of appropriate tables and graphics | We included the PRISMA flow-chart and several tables and graphics to describe the literature search and our results. |
| **Reporting of results should include** | |  |
| √ | Graph summarizing individual study estimates and overall estimate | We have appended them in the main text. Additional graphs were presented as supplementary material to fully describe the results. |
| √ | Table giving descriptive information for each study included | We have presented descriptive information for each study in the supplementary material. |
| √ | Results of sensitivity testing | Subgroup analyses were conducted regarding the different definitions of remission and recover, the type of included studies, and the period of follow-up. |
| √ | Indication of statistical uncertainty of findings | We reported mean estimates for the main outcome and 95% CI. |
| **Reporting of discussion should include** | |  |
| √ | Quantitative assessment of bias | Publication biases were assessed with Egger test [2]. |
| √ | Justification for exclusion | Exclusion criteria and justification are described in the manuscript. |
| √ | Assessment of quality of included studies | We adapted the Newcastle-Ottawa Scale for the evaluation of cross-sectional and cohort studies. |
| **Reporting of conclusions should include** | |  |
| √ | Consideration of alternative explanations for observed results | We discussed other explanations for our findings in the discussion section. |
| √ | Generalization of the conclusions | We have addressed the generalization of the conclusions in the discussion section. |
| √ | Guidelines for future research | We have suggested possible streams of future development and research in the discussion. |
| √ | Disclosure of funding source | Funding source described at the end of the manuscript. No separate funding was necessary for the undertaking of this meta-analysis. |

**Table S3**. Risk of bias (quality) assessment using modified Newcastle Ottawa Scale for cross-sectional and cohort studies.

| **Criteria** | **Maximum Score** |
| --- | --- |
| *Cross Sectional Studies* | |
| Sample representative of target sample (e.g. all eligible or random sample)? | 2 |
| Sample size justified and satisfactory? | 1 |
| Non-response rate is defined satisfactory, and characteristics of responders/non-responders compared? | 1 |
| Ascertainment of exposure (i.e. menstrual cycle) is valid and/or well described? | 1 |
| Assessment of outcome with robust tool and/or record linkage? | 2 |
| Outcome per group reported appropriately? | 1 |
| *Cohort Studies* | |
| Representativeness of exposed cohort (e.g. total population or random sample. selected group) | 1 |
| Method used to ascertain exposure (menstrual cycle phase) is robust? | 1 |
| Exposed and unexposed are matched or adjustment for confounding factors? | 2 |
| Assessment of outcome was blind to exposure status or used record linkage. were robust tools used? | 2 |
| Follow-up period was sufficiently long for outcomes to occur (e.g. more than one menstrual cycle? | 1 |
| Loss to follow-up rate is reported. low (<30%). and same in exposed and non-exposed? | 1 |

**Results S1.** Systematic review

Forty-two studies described remission predictors (Table S7) and 28 recovery predictors (Table S8). The most studied predictors were socio-demographic and clinical variables.

- 1. **Remission**

Twelve studies [3-14] found that socio-demographic variables, e.g. age, sex, years in education, and other socio-demographic variables were associated with remission status at follow-up. Thirteen studies described an association between DUP and remission. In ten studies, baseline psychotic symptoms or an early reduction in psychotic symptoms (positive, negative or general) were associated with later remission. Finally, five studies found associations between premorbid functioning [6, 10, 15] and premorbid social adjustment [16, 17] and remission.

One study found that exposure to at least one type of childhood adversity was significantly associated with a reduced remission rate[18]. Four studies found a significant association between cognitive function and remission. Boden et al. [19], showed that better performance on finger tapping with the non-dominant hand was associated with an increased risk of a 5-year symptomatic non-remission. Better short-term verbal memory [20] and attention at baseline[21] were also associated with remission. Another study found that better global cognitive functioning was associated with remission, adequate social/vocational functioning and full recovery criteria at 5-year follow-up [22].

Two studies [22-24] investigated neuroimaging and biochemistry in relation to remission. Jauhar et al. [23], showed a positive correlation between associative striatal baseline dopamine synthesis capacity and improvement in PANSS scale following antipsychotic treatment. In contrast, striatal volumes did not differ in patients who responded to antipsychotic treatment relative to those who did not and healthy controls. Brain volume measures at the whole-brain level, in the cortex, lateral and third ventricle, caudate, superior temporal gyrus and hippocampus were not associated with remission at 5-year follow-up[22]. Levels of serum brain-derived neurotrophic factor (BDNF) did not differ significantly over time and were not correlated with remission status at 6- and 12-month follow-up visits[24] in another study.

Two studies found that a significantly higher proportion of patients with a normal electroencephalogram (EEG) at baseline were in remission at 2-year follow-up (94.4%) compared to patients who had an essentially normal or dysrhythmic EEG (75.8%) [25].

Three studies found a significant association between the type of intervention and remission, all of which were RCTs. One found that in patients treated with Olanzapine as opposed to haloperidol, the remission rate was greater (57.25% vs 43.94%)[26]. Another reported that the proportion of patients in remission was higher for those treated with second-generation antipsychotics as compared to low doses of haloperidol[27]. A third study compared treatment with risperidone long-acting injectable (RLAI) and treatment with oral antipsychotics^[28]^. Although not statistically significant, the remission rate was higher in the RLAI group[28].

Malla eta al.[29] reported that patients managed by Early Intervention Services (EIS) (which provided case management, family intervention, cognitive behaviour therapy (CBT) and crisis intervention), had a significantly longer mean length of remission of both positive and negative symptoms compared to patients managed by generic services. In another RCT comparing specialised EIS and generic care, perceived emotional support significantly predicted longer time spent in remission[30].

- 1. **Recovery**

Sixteen studies [3, 6, 9, 11, 14, 21, 31-40] reported associations between socio-demographic variables and recovery, and eighteen identified clinical variables associated with recovery. Five studies found associations between recovery and premorbid functioning [37, 41] and premorbid social adjustment [16, 32, 42]. Speed of processing and executive functions at baseline were significant correlates of recovery at 3-year follow-up [42]. One study found that a composite index of cortical asymmetry was significantly associated with recovery [22].

An RCT that compared dose reduction/discontinuation of antipsychotics (DR) with maintenance treatment (MT) found that the former was linked to long-term recovery proportion and functional remission proportion, but not to symptomatic remission [40]. The OPUS trial compared EIS care (comprising intensive psychosocial assertive community treatment, psychoeducation, social skills training, family treatment and multifamily groups) with standard treatment and found that EIS care was more likely to be associated with full recovery [43].

**Table S4.** Remission/recovery criteria

| Remission | Symptoms remission as defined by the psychometric instruments (e.g., PANSS, CGI, BPRS, SAPS/SANS) |
| --- | --- |
| Recovery | Symptoms remission plus significant improvement in functioning. The latter being defined by the studies based on psychometric instruments (e.g., SOFAS, GAF) or improvements in functioning domains (e.g., employment/studies, living independent, social interactions) |

PANSS, Positive and Negative Syndrome Scale; CGI, Clinical Global Impression; BPRS, Brief Psychotic Rating Scale; SAPS/SANS, Scale for Assessment of Positive Symptoms/Scale for Assessment of Negative Symptoms; SOFAS, Social and Occupational Functioning Assessment Scale; GAF, Global Assessment of Functioning

**Table S5.** Description of studies with remission data

| **Author, year** | **Type of study** | **Country** | **Follow up (months)** | **N FEP**  **Baseline-FU** | **Age mean (SD)** | **Male (%)** | **Definition of remission** | **N (%)** | **NOS** |
| --- | --- | --- | --- | --- | --- | --- | --- | --- | --- |
| Addington, 2008[15] | Cohorts | Canada | 26.4 | 240-240 | 24.5 (17.2) | 82.08 | Remission criteria proposed by the Remission in Schizophrenia Working Group (RSWG) | 88 (36.7) | 5 |
| Ajnakina, 2018[18] | Cohorts | UK | 60 | 237-149 | 30.1 (10.3) | 62.9 | A continuous period of ≥6 months of a complete absence of a clear record of psychotic symptoms in clinical notes, including no evidence of re-emergence of psychotic symptoms, re-admission to psychiatric wards, and/or having been re-referred to acute home treatment/crisis intervention services during the follow-up period | 129 (54.4) | 5 |
| Alaghband-Rad, 2006[44] | Cohorts | Iran | 24 | 54-49 | 24.2 (8.9) | 46.3 | Being symptom free for at least 4 weeks | 15 (30.6) | 5 |
| Barrio, 2013[28] | Case-control | Spain | 24 | 26-26 | 26.9  (6.7) | 61.5 | Remission criteria proposed by RSWG | 17 (63) | 6 |
| Benoit,2014[20] | Canada | Cohorts | 12 | 70-70 | 23 (7.58) | 74.28 | Remission criteria proposed by RSWG | 17 (24.3) | 4 |
| Boter, 2009[27] | RCT | $\geq$13 European countries and Israel | 12 | 498-498 | 26 | 60 | Remission criteria proposed by RSWG | 151 (30.3) | 7 |
| Ceskova, 2008[45] | Cohorts | Czech Republic | 12 | 93-93 | 25.3 (5.45) | 100 | Remission criteria proposed by RSWG | 73 (78) | 4 |
| Ceskova, 2010[46] | Cohorts | Czech Republic | 84 | 44-44 | 22.2 (5.2) | 100 | Remission criteria proposed by RSWG | 23 (52.27) | 4 |
| Chang, 2012[47] | Cohorts | China | 36 | 539-539 | 21 (3.37) | 55.4 | CGI-S scores < 3 for positive and negative symptoms in the last 6 months | 317 (58.8) | 5 |
| Chang, 2013[4] | Cohorts | China | 24 | 73-73 | 25.8 (9.6) | 54.8 | Remission criteria proposed by RSWG | 61 (83.6) | 6 |
| Clausen, 2014[48] | Cohorts | Denmark | 60 | 578-314 | 26.5 (6.3) | 59.3 | Remission criteria proposed by RSWG | 172 (55.5) | 7 |
| Colizzi, 2016[5] | Cohorts | UK | 12 | 205-205 | 29.6  (6.6) | 63.40 | Psychiatric and Personal History Schedule (PPHS): Absence of positive, negative or disorganized symptoms for at least 30 days | 93 (45.4) | 5 |
| Conus, 2017[6] | Cohorts | Australia | 24 | 584-584 | 22.06 (3.4) | 66.09 | A score of no ≤ 3 on the all three of the discharge CGI rating scales | 383 (65.58) | 6 |
| Crespo-Facorro, 2011[49] | RCT | Spain | 36 | 174-174 | 27.4 (7.8) | 62 | Remission criteria proposed by RSWG | 54 (31.03) | 6 |
| Crumlis, 2009[50] | Cohorts | Ireland | 95.3 | 118-67 | 24.4 (6.5) | 73.1 | Score ≤3 on any of the 30 PANSS items over the previous month | 33 (49.25) | 5 |
| Dazzan, 2019[7] | Cohorts | UK | 120 | 387-303 |  | 55.6 | Remission criteria proposed by RSWG | 213 (65.3) | 4 |
| de Haan, 2008[51] | Cohorts | The Netherlands | 60 | 110-110 | 21.1 (2.8) | 84.54 | Remission criteria proposed by RSWG for 9 months | 41 (37.5) | 5 |
| De Lisi, 1998[52] | Cohorts | USA | 60 | 50-50 | 27.4 (7) | 64 | Severity of illness scores of 0-1 on BPRS was considered ‘remission; and ‘partial remission was based on a decrease in the peak severity score of at least two points | 5 (10) | 5 |
| Drake, 2014[53] | RCT | UK | 10.5 | 61-61 | 24.06 (4.8) | 60.65 | Remission of symptoms was defined as PSYRATS score of zero | 16 (26.23) | 7 |
| Emsley, 2007[54] | RCT | Multicenter | 24-48 | 462-462 | 25.27 (6.71) | 72 | Remission criteria proposed by the RSWG was considered as achieved at any  stage in the trial, regardless of whether participants were able to maintain this status for longer than 6 months until study endpoint | 109 (23.6) | 5 |
| Evensen, 2012[55] | Cohorts | Scandinavian countries | 120 | 301-186 | 27.59 (9.5) | 61.96 | Remission criteria proposed by RSWG | 92 (49.46) | 4 |
| Fraguas, 2014[56] | Cohorts | Spain | 24 | 110-41 | 17.78 (2) | 72.3 | Remission criteria proposed by RSWG | 25 (53.2) | 4 |
| Gaebel, 2007[57] | RCT | Germany | 24 | 151-151 | 31.6 (10) | 58.3 | Remission criteria proposed by RSWG | 38 (57,6) | 7 |
| Gaebel, 2014[8] | RCT | Germany | 12 | 166-166 | 31.8 (10) | 59.6 | Remission criteria proposed by RSWG | 65 (39.2) | 5 |
| Gasquet, 2008[58] | Cohorts | France | 36 | 563-563 | 37.5 (11) | 62.5 | A score of ≤3 on the CGI-S (overall, positive, negative and cognitive symptoms score), and maintained for $\geq$6 months. In addition, no hospitalization because of schizophrenia during 6 months | 341 (60.6) | 3 |
| Golay, 2019[9] | Cohorts | Switzerland | 36 | 257-257 | 24.55 (4.83) | 65.4 | Remission criteria proposed by RSWG | 75 (29.2) | 3 |
| Green, 2006[26] | EU/USA | Case-control | 24 | 263-263 | 23.76 (4.75) | 81.8 | No rating >3 on items P1, P2, P3, P5 and P6 of the PANSS; and CGI severity score ≤3 for at least 4 consecutive weeks | 133 (50.6) | 5 |
| Hassan, 2011[59] | Cohorts | Saudi Arabia | 24 | 56-37 | 17 (3.7) | 37.84 | Remission criteria proposed by RSWG | 19 (51.4) | 4 |
| Hill, 2012[60] | Cohorts | Ireland | 120 | 171-123 | 29 (12) | 57.9 | Remission criteria proposed by RSWG | 74 (60.2) | 7 |
| Ho, 2000[61] | Cohorts | US | 6 | 74-74 | 24.7 (5.8) | 62.2 | A rating of ≤2 on all global scores within the SAPS psychotic and disorganized symptom dimensions persisting for at least 8 consecutive weeks | 43 (58.1) | 6 |
| Jablensky, 1992[62] | Cohorts | Multicenter | 24 | 1379-1078 |  | 54.02 | A score of ≤3 on the CGI-S maintained for $\geq$6 months | 317 (29.4) | 4 |
| Jauhar, 2019[23] | Case-control | UK | 6 | 26-26 | 25.31 (4.61) | 84.6 | Remission criteria proposed by RSWG | 14 (53.8) | 7 |
| Johnson, 2014[63] | Case-control | India | 60 | 131-9 | 29.5 (7.2) | 55 | Remission criteria proposed by RSWG | 65 (68.4) | 7 |
| Kaminga, 2018[10] | Cohorts | Malawi | 18 | 126-98 | 30.9 (10.8) | 67.3 | Remission criteria proposed by RSWG | 70 (71.4) | 6 |
| Kinoshita, 2005[64] | Cohorts | Japan | 180 | 97-52 | 24.8 (7.82) | 57.7 | No symptoms or signs of a psychotic episode for at least 4 weeks but having residual symptoms, non-psychotic symptoms or a character change | 17 (32.7) |  |
| Klaerke, 2019[34] | Cohorts | Denmark | 48-216 | 70-56 | 26.5 (6.2) | 71 | Remission criteria proposed by RSWG | 24 (34) | 5 |
| Lambert, 2006[65] | Cohorts | Germany | 24 | 2960-2905 | 42.3 (13.9) | 49.4 | CGI-S score of ≤ 3 in assessments of overall severity and positive, negative, and cognitive subscores for at least 6 months | 1397 (47.2) | 3 |
| Langeveld, 2012[66] | Cohorts | Norway | 24 | 232-187 | 25.16 (8.72) |  | PANSS score $\leq$3 on positive subscale items 1,3,5 or 6 or on general subscale 9 for at least one week | 149 (79.7) | 6 |
| Lieberman, 1992[67] | Cohorts | USA | 60 | 92-70 | 24.3 (6) | 55.7 | No rating >3 on any of the SADS-C[68], positive psychotic symptoms items, a CGI severity item rating of <3,or a CGI improvement item rating of $\geq$2 for 8 weeks | 59 (84) | 6 |
| Luckhoff, 2019[69] | Cohorts | South Africa | 12 | 106-98 |  | 72.64 | Remission criteria proposed by RSWG | 63 (64) | 4 |
| Malla, 2017[29] | RCT | Canada | 60 | 110-82 | 21.9 (4.1) | 68.2 | Patients scoring ≤2 on all of the global (subscale) items of either scale (SAPS, SANS) | 92 (41.8) | 7 |
| Mojtabai, 2003[70] | Cohorts | USA | 36 | 16-13 | 36.1 (13.1) |  | Remission was defined as no psychotic symptoms within 3 months, and between 3 and 6 months using the SAPS and SANS | 6 (46) | 5 |
| Manchanda, 2005[25] | Canada | Cohorts | 24 | 91-91 | 24.7 |  | Rating of 0 on all subscales of SAPS by the end of 1 year and maintaining this rating at 2-year follow-up; no period of recurrence of positive symptoms between 1 and 2 years based on consensus rating of independent chart review using a modified version of the Life Chart Schedule | 65 (42.1) | 4 |
| Mustafa, 2018[71] | Cohorts | Canada | 12 | 390-390 | 23.4 (4.5) | 72 | Score $\leq$ 2 on each of the global subscales on the SAPS for at least 1 month | 195 (50) | 7 |
| Nishida, 2018[72] | RCT | Japan | 18 | 77-68 | 23 (5.1) | 55.84 | Remission criteria proposed by RSWG | 33 (48.53) | 5 |
| Norman, 2014[73] | RCT | Canada | 60 | 225-132 | 26.36 (7.9) | 77.27 | Remission criteria proposed by RSWG | 110 (83.6) | 4 |
| O’Keeffe, 2019[16] | Cohorts | Ireland | 240 | 171-80 |  | 57.9 | Remission criteria proposed by the RSWG, without the prerequisite of 6 months stable state | 31 (86.1%) | 5 |
| Perkins, 2004[74] | Cohorts | Multicenter | 24 | 191-72 | 24 (4.9) | 80.01 | No rating >3 on items P1, P2, P3, P5, and P6 of the PANSS; and a CGI Severity score $\leq$3 for 4 consecutive weeks | 42 (62.3) | 6 |
| Petersen, 2008[43] | Cohorts | Denmark | 24 | 547-369 | 26.75 (6.22) | 58.3 | A rating of $\leq$ 3 on any global scores of SAPS and SANS at a single time point evaluation covering the previous month | 202 (26) | 6 |
| Renwick,2015[12] | Cohorts | Ireland | 12 | 375-215 | 32.9 (11.9) | 58.4 | Remission criteria proposed by RSWG during one month | 85 (39.53) | 6 |
| Robinson, 2004[22] | Cohorts | USA | 72 | 118-118 | 25.2 (6.6) | 52 | Rating of $\leq$ 3 for all of the following SADS-C psychosis items: severity of delusions, severity of hallucinations, impaired understandability, derailment, illogical thinking, and bizarre behaviour, and a rating of $\leq$3 for the SANS global ratings of affective flattening, alogia, avolition apathy, and anhedonia-asociality for 2 years or longer | 39 (47.2) | 5 |
| Saravanan, 2010[75] | Cohorts | India | 6 | 131-131 | 29.5 (7.2) | 55 | No positive or negative symptoms for at least 30 days | 58 (44.27) | 6 |
| Schennach-Wolff, 2011[76] | RCT | Germany | 8 | 224-132 | 31.44 (10.4) | 56.81 | Remission criteria proposed by RSWG | 132 (59) | 7 |
| Schennach, 2013[77] | RCT | Germnay | 12 | 132-132 | 31,43 (10.6) | 60 | Remission criteria proposed by RSWG | 99 (75) | 6 |
| Simonsen, 2017[78] | Cohorts | Norway | 12 | 254-254 | 27.5 (7.5) | 63 | Remission criteria proposed by RSWG | 24 (26) | 5 |
| Simonsen, 2010[79] | Cohorts | Denmark | 24 | 293-245 | 28.3 (9.8) | 55.5 | PANSS positive subscale items 1, 3, 5 or 6 score <4, and PANSS general subscale item 9 for at least 1 week | 245 (83.6) | 5 |
| Stralin, 2019[80] | Cohorts | Sweden | 12 | 175-148 | 28.7 | 55 | Remission criteria proposed by the RSWG, without the prerequisite of 6 months stable state | 101 (68.24) | 5 |
| Takeuchi, 2019[81] | Cohorts | Canada | 12 | 130-130 | 22.1 (3.37) | 86.9 | CGI-I score ≤2 and BPRS 4 score psychotic items scores ≤3 | 63 (48.46) | 6 |
| Tang, 2014[82] | Cohorts | China | 156 | 153-96 | 31.7 (9.2) | 45.75 | Remission criteria proposed by RSWG | 45 (47) | 6 |
| Tempier, 2013[30] | Cohorts | UK | 6 | 123-123 | 26,3 (6.1) | 36 | No psychotic symptoms in two consecutive months | 114 (92.7) | 6 |
| Thara, 2004[83] | Case-control | India | 240 | 90-61 | 24.5 | 50 | Absence of all positive and negative symptoms for a period of at least 1 month (relapses between episodes are possible) | 29 (47.5) | 3 |
| Torgalsbøen, 2014[84] | Cohorts | Norway | 6 | 28-17 | 21 (2.6) | 60.7 | Remission criteria proposed by RSWG | 17 (61) | 6 |
| Torgalsbøen, 2015[21] | Cohorts | Norway | 24 | 28-17 | 21.1 (2.6) | 60.7 | Remission criteria proposed by RSWG | 16 (64) | 6 |
| Ücok, 2011[13] | RCT | Turkey | 24 | 153-94 | 21.02 (4.78) | 52.13 | Remission criteria proposed by RSWG | 56 (59.5) | 5 |
| Valencia, 2012 | Cohorts | Mexico | 12 | 88-73 | 24.31 (3.1) | 74.32 | Remission criteria proposed by RSWG | 57 (78.1) | 6 |
| Vazquez-Barquero, 1999[85] | Cohorts | Spain | 36 | 86-76 |  | 43.02 | Absence of all psychotic symptoms using SANS and SAPS | 24 (31.5) | 6 |
| Verma, 2012[14] | Cohorts | Singapore | 24 | 1175-1175 | 28 (6.5) | 51.3 | Remission criteria proposed by RSWG | 636 (54.1) | 4 |
| Wade, 2006[86] | Cohorts | Australia | 15 | 126-103 | 21.6 (3.5) | 71 | BPRS 4 core psychotic items (conceptual disorganization, hallucinatory behaviour, suspiciousness, unusual thought) scores ≤3 for at least 2 weeks | 98 (95.14) | 6 |
| Wunderink, 2007[87] | RCT | The Netherlands | 18 | 125-125 | 26.4 (6.4) | 68.8 | Remission criteria proposed by RSWG | 60 (48) | 4 |
| Wunderink, 2008[88] | RCT | The Netherlands | 24 | 125-125 | 26.4 (6.4) | 68.8 | Remission criteria proposed by RSWG | 65 (52) | 4 |
| Wunderink, 2013[40] | RCT | The Netherlands | 84 | 103-52 | 26.26 (6.79) |  | Remission criteria proposed by RSWG | 36 (69.2) | 4 |
| Xenaki, 2020[89] | Cohorts | Greece | 12 | 130-93 | 26 | 64.6 | Remission criteria proposed by RSWG | 53 (54.7%) | 4 |
| Yee, 2019[24] | Case-control | Singapore | 12 | 29-29 | 28.8  (9.8) | 44.8 | Remission criteria proposed by RSWG | 11 (37.9) | 3 |
| Zarate, 2000[90] | Cohorts | USA | 24 | 30-22 | 27.5 (9.4) | 63.3 | Operational criteria of recovery were based on features that initially established a DSM-III-R diagnosis, rated weekly on a severity scale from 1 to 7, that followed the definitions of the CGI scale. In addition, specific operational criteria for recovery, for schizophrenia and schizophreniform disorder, were: no DSM-III-R ‘A’ criteria rated >2, and fewer than three criteria rated $\geq2$. Furthermore, a minimum period of 8 weeks of the above criteria had to be completed for a subject to be considered | 19 (86.4) | 3 |
| Zhang, 2016[91] | Cohorts | China | 24 | 347-99 | 26.2  (6.3) | 23.8 | A CGI-S score $\leq$3 | 265 (76.4) | 6 |
| Zhou, 2017[92] | Cohorts | China | 12 | 40-32 | 26.2 (8.1) | 59.4 | Remission criteria proposed by the RSWG, without the prerequisite of 6 months stable state | 19 (59.37) | 4 |

BPRS, Brief Psychiatric Rating Scale; CGI-S, Clinical Global Impression Scale; GAF, Global Assessment of Functioning; PANSS, Positive and Negative Syndrome Scale; PSYRATS, Psychotic Symptoms Rating Scale; SADS-C, Schedule for Affective Disorders and Schizophrenia; SANS, Scale for Assessment of Negative Symptoms; SAPS, Scale for Assessment of Positive Symptoms

**Table S6.** Description of studies with recovery data.

| **Author, year** | **Type of study** | **Country** | **Follow up (months)** | **N FEP**  **Baseline-FU** | **Age mean (SD)** | **Male (%)** | **Definition of recovery** | **N (%)** | **NOS** |
| --- | --- | --- | --- | --- | --- | --- | --- | --- | --- |
| Ajnakina, 2018[18] | Cohorts | England | 60 | 237-149 | 30.1 (10.3) | 62.9 | Recovery as remission sustained for ≥2 years | 107 (45.15) | 5 |
| Austin, 2013[31] | Cohorts | Denmark | 120 | 304-262 | 37,19  (6.48) | 55.3 | Symptomatic remission, no hospitalization, or living in supporting accommodation for the past 2 years, currently engaged in work or study, GAF>60 | 43 (14) | 6 |
| Álvarez-Jiménez, 2012[32] | Cohorts | Australia | 120 | 209-209 | 21.9 (3.5) | 44 | According to the QLS: appropriate interpersonal relationships with people outside the family (item 4 ≥4); adequate vocational functioning defined as paid employment, attending school or, if a homemaker, performing that role efficiently (item 9 ≥4) and adequate accomplishment, defined as success in fulfilling the particular role that the person has chosen to attempt (item 10≥4); regular participation in basic living tasks (item 19≥4) | 54 (26%) | 6 |
| Bachmaan, 2008[93] | Cohorts | Germany | 12 | 62-40 | 27.4 (8.1) | 100 | Symptomatic and functional recovery was defined according to a GAF score ≥61 during the month prior to assessment | 27 (67.5) | 5 |
| Bertelsen, 2009[94] | Cohorts | Denmark | 24 | 265-265 | 26 (5.8) | 58 | Combination of both symptomatic remission, according to the RSWG, and functioning of >59 on GAF scale, employed or studying during the previous year | 45 (17) | 4 |
| Bjornestad, 2017[33] | Cohorts | Norway | 24 | 178-178 | 26.85 (10.73) | 53.9 | Social functioning was measured by the subscales measuring work and social interaction (SCLFS) and the criteria of living independently (LQoLI) day-to-day living (independent living), role functioning (work, academic, or full-time homemaking), and social interaction. A score of 0 indicated very poor functioning and>3 indicated adequate functioning. Participants had to have fulfilled these criteria for the las consecutive 12 months to obtain scores>3 on the SCS. Clinical recovery was operationalized as a single variable of “yes” for all patients who met criteria for both symptom (RSWG) and functional remission | 24 (40) | 6 |
| Boden, 2014[19] | Cohorts | Sweden | 60 | 46-40 | 27 (8) | 63 | Combination of both symptomatic remission (RSWG) and functioning (5-point Likert scale). Occupational and social function was assessed using the Strauss-Carpenter Functioning Scale, independent living | 14 (35) | 5 |
| Chang, 2012[47] | Cohorts | China | 36 | 539-539 | 21 (3.37) | 55.4 | Fulfillment of the following criteria in the last 12 months of study period: CGI-S scores < 3 for both positive and negative symptoms; no psychiatric admission; achieving employment (full or part-time work/study), and SOFAS<60 | 94 (17.4) | 5 |
| Conus, 2017[6] | Cohorts | Australia | 24 | 584-584 | 22.06 (3.4) | 66.09 | Having a regular activity based on MVSI; and independent living according to the Modified Location Code Index (i.e., head of household, living alone, with partner, or with peers, and living with family with minimal supervision) | 230 (39.38) | 6 |
| Craig, 2000[95] | Cohorts | USA | 23 | 149-149 |  | 66.5 | Residual psychopathological symptoms of insignificant severity (PANSS item scores of ≤3) may be present but no evidence of significant effect on "functioning" | 21 (14.1) | 5 |
| Evensen, 2012[55] | Cohorts | Multicenter | 120 | 301-186 | 27.59 (9.5) | 61.96 | Combination of symptomatic remission and good functioning using the SCLFS (scores>4): independent living, role functioning (employed or studying)and social interaction | 45 (24.2) | 4 |
| Faber, 2012[96] | RCT | The Netherlands | 24 | 124-124 | 25.7 (6.7) |  | Combination of both symptomatic remission and improved functioning using the GSDS: no score higher than 2 or on GSDS role scale, no functional relapses | 32 (25.7) | 4 |
| Golay, 2019[9] | Cohorts | Switzerland | 36 | 257-257 | 24.55  (4.83) | 65.4 | GAF$\geq$60, independent living and work at the last assessment of the last year of the program | 40 (15.5) | 3 |
| González-Blanch, 2010[97] | Cohorts | Spain | 24 | 171-131 | 26 | 62 | Currently employed or studying, with the same or better level of performance as before the psychotic episode and with a minimal social disability (scores of 0 or 1 in the DAS) | 34 (25.95) | 6 |
| Harrison, 2001[98] | Cohorts | Multicenter | 180 | 1005-1005 |  |  | Using a modified version of Bleuler's typology, which combines mode of onset (acute versus insidious), overall trajectory (simple versus episodic), and end state (Bleuler recovered or minimal symptomatology (good), versus moderate or severe impairment (poor). Bleuler ratings of recover and GAF disability>60 | 441 (43.9) | 4 |
| Harrow, 1997[99] | Case-control | USA | 90 | 276-74 | 23.1 | 51 | Absence of psychotic symptoms (delusions, hallucinations, formal thought disorder or catatonia) for at least 1 month with the total score on the BPRS lower than 10 and GAF score >70 at the last interview | 23 (32) | 4 |
| Harrow, 2005[100] | Case-control | USA | 180 | 274-157 | 28.8 | 67 | Absence of major symptoms, and adequate psychosocial functioning, including being employed (score $\geq$2 on SCS), absence of a very poor social activity level (score $\geq$ 2 on the S-C Social Activity Scale), no hospitalizations during the follow-up year | 67 (42.7) | 4 |
| Klaerke, 2019[34] | Cohorts | Denmark | 48-216 | 70-56 | 26.5 (6.2) | 71 | Combination of both symptomatic and functional remission according to Lieberman et al.[101] | 16 (23) | 5 |
| Kurihara, 2011[102] | Cohorts | Indonesia | 204 | 59-43 |  | 58.13 | Combination of both symptomatic remission (RSWG and good functioning: (1) vocational functioning: employed, (2) independent living (3) peer relationships: have meetings with nonfamily members more than once per week for 6 months | 14 (23.7) | 5 |
| Lambert, 2006[65] | Cohorts | Germany | 24 | 2960-2905 | 42.3 (13.9) | 49.4 | Positive occupational/ vocational status: employed or studying, independent living | 787 (26.6) | 3 |
| Mattsson, 2008[103] | Cohorts | Sweden | 60 | 71-71 |  | 47.9 | GAF score stable at $\geq$60 for at least 6 months, employed or studying | 52 (73.24) | 5 |
| O’Keeffe, 2019[16] | Cohorts | Ireland | 240 | 171-80 |  | 57.9 | Combination of both symptomatic remission (RSWG) and functional and vocational recovery status evaluated by the QLS | 25 (32.5) | 5 |
| Petersen, 2008[43] | Cohorts | Denmark | 24 | 547-369 |  | 58.3 | Combination of both symptomatic remission and social and (or) vocational functioning: employed or studying, living independently, no hospitalization during the preceding year | 102 (15) | 6 |
| Phahladira, 2020[37] | Cohorts | South Africa | 24 | 98-98 | 24.2 (6.4) | 73 | Combination of both symptomatic remission (RSWG) and good functioning, defined as a SOFAS score $\geq$61; QLS score of 4 or 5 | 28 (28.57) | 6 |
| Rangaswamy, 2012[104] | Cohorts | India | 24 | 47-39 | 29.74 | 8.7 | PANSS score $\leq$60 and a GAF > 80 | 28 (71.8) | 5 |
| Rangaswamy, 2012a[105] | Cohorts | India | 300 | 90-47 | 24.5 |  | Recovery without relapse | 7 (15) | 3 |
| Robinson, 2004[22] | Cohorts | USA | 60 | 118-118 | 25.2  (6.6) | 52 | Combination of both symptom remission and adequate social/vocational functioning: (1) employed or studying, (2) performing day-to-day living tasks without supervision, (3) social interactions once a week or more with friends or romantic contacts for ≥2 years | 12 (13.7) | 5 |
| Simonsen, 2017[78] | Cohort | Norway | 12 | 254-67 | 27.5 (7.5) | 63 | Combination of both symptomatic remission and good function t 1-year follow-up, as defined by a GAF score ≥61, employed or studying for at least 50%, living independently | 13 (14) | 5 |
| Shrivastava, 2010[38] | Cohorts | India | 120 | 200-101 | 28.8 (8.2) | 73.3 | Complete symptomatic remission and total social integration | 61 (60.4) | 3 |
| Sullivan, 2018[106] | Cohorts | UK | 12 | 2134-2134 | 23.2 (4.8) | 50 | Validated assessment of recovery according to Bebbington et al., 2005[107] | 983 (46) | 4 |
| Tarricone, 2014[108] | Cohorts | Italy | 12 | 163-135 | 31 (9.4) | 56 | Recovery according to the Selten et al. (2007)[109] criteria (absence of psychiatric symptoms and function on the pre-morbid level) | 4 (2) | 5 |
| Thorup, 2014[39] | RCT | Denmark | 60 | 578-311 |  | 45 | Symptomatic remission during the last 2 years, good functioning as defined by a GAF score >60, employed or studying, no hospitalization or living in a supported housing facility during the last 2 years | 54 (17.4) | 5 |
| Torgalsbøen, 2015[21] | Cohorts | Norway | 24 | 28-25 | 21.1 (2.6) | 60.7 | Combination of symptomatic remission (RSWG) and the operational recovery criteria developed by Liberman et al. (2002) concerning psychosocial functioning: (1) at least part-time work or school, (2) living independently, (3) at least once weekly socializing with peers or otherwise involved in recreational activities that are age-appropriate and independent of professional supervision. GAF: Social and Role score of 8 (adequate social/interpersonal functioning and good role functioning) | 4 (16) | 6 |
| Treen Calvo,2018[42] | Cohorts | Spain | 36 | 577-399 | 30.1 (9.5) | 57.89 | Minimal or no symptoms and a return to a premorbid functional state | 113 (28.32) | 6 |
| Valencia, 2012[110] | RCT | Mexico | 12 | 88-73 | 24.31 (3.1) | 74.32 | Remission according to the Torgalsbøen[111] criteria with a GAF > 65 | 23 (31.51) | 6 |
| van Os,1996[112] | Cohorts | UK | 48 | 191-166 | 26.4 (6.5) | 44 | Symptom-free and shows his/her usual pre-morbid personality in 2 years | 75 (45) | 7 |
| Verma, 2012[14] | Cohorts | Singapore | 24 | 1175-1175 | 28 (6.5) | 51.3 | Combination of both symptomatic remission and good functioning, as defined by a GAF disability score $\geq$61, and employed or studying at 2 years | 345 (29.4) | 5 |
| Wunderink, 2008[88] | RCT | The Netherlands | 24 | 125-125 | 26.4 (6.4) | 68.8 | Combination of both symptomatic remission and functioning for at least 6 months at the 7-year follow-up | 33 (26.4) | 4 |
| Wunderink, 2013[40] | RCT | The Netherlands | 84 | 103-52 | 26.26 (6.79) |  | Criteria for recovery were met when patients had symptomatic (RSWG) and functional remission for at least 6 months at the 7-year follow-up | 21 (40.4) | 4 |

GAF, Global Assessment of Functioning; QLS, Quality of Life Scale; SCLFS, Strauss Carpenter Level of Functioning Scale; LQoLI. Brief Version of Lehman's Quality of Life Interview; CGI-S, Clinical Global Impression Scale; MVSI, Modified Vocational Status Index; BPRS, Brief Psychotic Rating Scale; GSDS, Groningen Social Disabilities Schedule; DAS, Disability Assessment Schedule; SAPS, Scale for Assessment of Positive Symptoms; SANS, Scale for Assessment of Negative Symptoms; PANSS, Positive and Negative Syndrome Scale; SOFAS, Social and Occupational Functioning Assessment Scale; SAS, Social Adjustment Scale; DSM, Diagnostic and Statistical Manual Mental

**Table S7.** Predictors of studies with remission data

| **Author, year** | **Investigated predictors (N)** | **Predictors associated with remission (N)** | **Predictor categories associated with remission** | | | | | |
| --- | --- | --- | --- | --- | --- | --- | --- | --- |
|  |  |  | **Socio-demographic** | **Clinical** | **Pre-morbid** | **Cognitive** | **Neuroimaging & bio-chemistry** | **Intervention** |
| Addington, 2008[15] | Insight, GAF, positive and negative symptoms, quality of life, premorbid functioning, DUP, change in positive symptoms, change in negative symptoms, cannabis use (10) | Positive and negative symptoms, quality of life, premorbid functioning, change in positive symptoms, change in negative symptoms (6) |  | x | x |  |  |  |
| Ajnakina, 2018[18] | Sexual abuse, physical abuse, parental separation, parental loss, institutional loss, institutional care, family arrangements, total adversity | Total adversity more than 1 (1) |  | x |  |  |  |  |
| Barrio, 2013[28] | Risperidone long-acting injectable (1) | Not significant |  |  |  |  |  |  |
| Benoit,2014[20] | Verbal memory, visual memory, and working memory (3) | Verbal memory (1) |  |  |  | x |  |  |
| Boden, 2014[19] | Cognitive variables | Finger tapping test (1) |  |  |  | x |  |  |
| Boter, 2009[27] | Haloperidol, amilsupride, olanzapine, quetiapine, adherence with antipsychotic medication, substance misuse (6) | Amilsupride, olanzapine, adherence with antipsychotic medication, substance misuse (4) |  | x |  |  |  | x |
| Chang, 2012[47] | Sex, age, educational level, employment status, SOFAS, age at onset, DUP, diagnosis, substance abuse, CGI-S positive and negative symptoms, symptom remission within first 3 months of treatment, number of hospitalisations, length of hospitalisations, suicide attempt, antipsychotic medication (16) | Sex, age at onset, DUP, symptom remission within first 3 months of treatment (4) | x | x |  |  |  |  |
| Chang, 2013[4] | Not stated | Educational level, baseline PANSS negative symptoms, symptomatic remission status at 1 year, disability assessment schedule (4) | x | x |  |  |  |  |
| Colizzi, 2016[5] | Nicotine dependence, problem drinking, cannabis use, stimulants use, poor medical adherence (5) | Nicotine dependence, problem drinking, cannabis use, stimulants use, poor medical adherence (5) | x | x |  |  |  |  |
| Conus, 2017[6] | Age, sex, diagnosis, CGI severity of illness, CGI depression score, CGI mania score, employment status, education, insight, family history of psychotic disorders, premorbid functioning, traumatic events in the past, age at onset, DUP, duration of untreated prodrome, suicide attempt, forensic history, substance use disorder, past psychiatric disorder (19) | Years in school, premorbid functioning, employment status (3) | x | x | x |  |  |  |
| Crumlish, 2009[50] | Age, sex, PSA, years in education, lifetime history of substance misuse, duration of prodrome, DUP, negative symptoms, positive symptoms, disorganized symptoms, insight, GAF (12) | DUP (1) |  | x |  |  |  |  |
| Dazzan, 2019[7] | Age, sex, ethnicity, index of social disadvantage (e.g., living alone), loss of parent, level of education, remission at 12 weeks, mode of onset insidious diagnosis, DUP, symptom severity (11) | Ethnicity others, index of social disadvantage (e.g., living alone), remission at 12 weeks, mode of onset insidious, diagnosis (5) | x | x |  |  |  |  |
| Drake, 2014[53] | Self-esteem (1) | Self-esteem (1) |  | x |  |  |  |  |
| Emsley, 2007[54] | Age, sex, age at onset, BMI, previous antipsychotic treatment, DUP, premorbid adjustment, antipsychotic medication dose, PANSS factor scores (9) | DUP, early clinical response (defined as >20% reduction of PANSS symptoms at 6 weeks) (2) |  | x |  |  |  |  |
| Fraguas, 2014[56] | DUP, age, gender, baseline GAF, baseline total PANSS score, cumulative antipsychotic doses (chlorpromazine equivalents), PAS score, IQ (9) | GAF, DUP (2) |  | x |  |  |  |  |
| Gaebel, 2014[8] | Age, sex, substance abuse, age at onset, functioning, baseline PANSS positive symptoms, negative symptoms, general psychopathology, depression, GAF, compliance, antipsychotic side effects (11) | Sex, functioning, baseline PANSS positive symptoms, negative symptoms, general psychopathology, compliance (6) | x | x |  |  |  |  |
| Gasquet, 2008[58] | Not stated | Never treated with antipsychotic medication, symptom severity type of antipsychotic medication (3) |  | x |  |  |  |  |
| Golay, 2019[9] | Migration (1) | Migration (1) | x |  |  |  |  |  |
| Green, 2006[26] | Antipsychotic medication: olanzapine versus haloperidol (1) | Antipsychotic medication: olanzapine versus haloperidol (1) |  |  |  |  |  | x |
| Hassan, 2011[59] | Age at onset, PAS, negative symptoms, compliance (4) | None |  |  |  |  |  |  |
| Hill, 2012[60] | Age, gender, PSA, years in education, lifetime substance misuse, DUI, DUP, diagnosis, baseline positive symptoms, negative symptoms, disorganized symptoms, insight, GAF (13) | DUP (1) |  | x |  |  |  |  |
| Jauhar, 2019[23] | Age, DUP, antipsychotic use, substance use, striatal volumes, dopamine synthesis capacity (6) | Dopamine synthesis capacity in associative striatum (1) |  |  |  |  | x |  |
| Kaminga, 2018[10] | Age at onset, sex, baseline PANSS positive symptoms, negative symptoms, general psychopathology, premorbid functioning, insight, marital status, education, vocational level, DUP, GAF, family history of psychosis (13) | Sex, marital status, DUP, Premorbid functioning, GAF (5) | x | x | x |  |  |  |
| Klaerke, 2019[34] | Age, sex, age at onset, cohort according year of inclusion (4) | Cohort (1) |  | x |  |  |  |  |
| Lambert, 2006[65] | Age, sex, DUI, CGI severity, baseline PANSS positive symptoms, negative symptoms, general psychopathology, vocational status, independent living, subjective well-being, health related quality of life, treatment with antipsychotics before baseline, type of initial antipsychotic medication, side effects, substance use disorder, compliance (16) | CGI overall score, CGI positive subscore, CGI negative subscore, vocational status, independent living, treatment with antipsychotics before baseline, type of initial antipsychotic medication, early symptomatic remission, early subjective well-being, substance use disorder, compliance (11) | x | x |  |  |  |  |
| Malla, 2017[29] | Type of intervention (1) | Type of intervention (1) |  |  |  |  |  | x |
| O’Keeffe, 2019[16] | Premorbid adjustment, diagnosis, employment, GAF, living alone, lifetime use of alcohol, DUP (7) | Premorbid social adjustment (1) |  |  | x |  |  |  |
| Petersen, 2008[43] | Age, sex, DUP, academic dimension, social dimension, psychotic dimension, negative dimension, substance use, vocational education, adherence to medication, type of treatment | DUP, baseline PANSS negative symptoms, medication adherence (3) |  | x |  |  |  |  |
| Renwick, 2015[12] | Sex, age, premorbid adjustment, duration of prodrome, DUP, baseline positive symptoms, negative symptoms, irritability (8) | Sex, DUP, positive symptoms (3) | x | x |  |  |  |  |
| Robinson, 2004[22] | Age, sex, ethnicity, education, social class, duration of psychosis symptoms at entry, diagnosis, positive symptoms, negative symptoms, premorbid social adjustment, cognitive domains: language, memory, attention, executive function, motor function, visuospatial function, premorbid cognitive functioning, social adjustment, MRI brain volumes, time taking antipsychotic medication (20) | Duration of psychosis, symptoms at entry, cognition (global), diagnosis (4) |  | x |  | x |  |  |
| Saravanan, 2010[75] | Age, sex, marital status, DUP, education, BPRS score, insight (7) | DUP, insight (2) |  | x |  |  |  |  |
| Schennach-Wolff, 2011[76] | Antipsychotic pre-treatment, early response, medication side effects, DUP, PANSS total score (5) | Early response, DUP, baseline PANSS total score (3) |  | x |  |  |  |  |
| Simonsen, 2017[78] | DUP, PANSS positive symptoms, negative symptoms, disorganized symptoms, GAF, social functioning during childhood, neurocognitive domains: current IQ, verbal learning and memory, processing speed, working memory, verbal fluency, interference control (12) | DUP, PANSS positive symptoms (2) |  | x |  |  |  |  |
| Simonsen, 2010[79] | Age, sex, relationship status, premorbid social functioning, DUP, diagnosis (6) | DUP (1) |  | x |  |  |  |  |
| Tang, 2014[82] | DUP, education, PAS social subscale, PAS academic subscale, schizophrenia diagnosis, smoking, PSST (7) | DUP, premorbid social adjustment |  | x | x |  |  |  |
| Tempier, 2013[30] | Perceived support (1) | Perceived support (1) |  |  |  |  |  | x |
| Torgalsbøen, 2014[84] | Speed of processing, attention and vigilance, working memory, verbal learning, visual learning, reasoning/problem solving, social cognition, MATRICS overall components (8) | Attention and vigilance, verbal learning (2) |  |  |  | x |  |  |
| Ücok, 2011[13] | DUP, duration of follow-up, total SANS at three-month, total PAS at childhood, adherence to medication, occupational status (10) | Adherence to medication, occupational status (2) | x | x |  |  |  |  |
| Verma, 2012[14] | Age, sex, marital status, education, ethnicity, diagnosis, early improvement at month 3, DUP, baseline PANSS positive symptoms, negative symptoms, general psychopathology, GAF (12) | Sex, educational status, early improvement at month 3, DUP, PANSS negative symptoms, PANSS general psychopathology, GAF disability (7) | x | x |  |  |  |  |
| Wunderink, 2008[88] | Discontinuation of antipsychotics versus maintenance treatment (1) | Not significant |  |  |  |  |  |  |
| Wunderink, 2013[40] | DUP, social functioning, living alone, treatment: dose reduction/discontinuation of antipsychotics (DR) versus maintenance treatment (MT) (4) | Social functioning, treatment: dose reduction/discontinuation of antipsychotics (DR) versus maintenance treatment (MT) (2) |  | x |  |  |  | x |
| Yee, 2019[24] | Levels of serum brain-derived neurotrophic factor (BDNF) (1) | Not significant |  |  |  |  |  |  |

BMI, body mass index; BPRS, Brief Psychiatric Rating Scale; CGI, Clinical Global Impression; DUI, duration of untreated illness; DUP, duration of untreated psychosis; GAF, Global Assessment of Functioning; PANSS, Positive and Negative Symptom Scale; PAS, Premorbid Adjustment Scale; PSST, Premorbid Schizoid and Schizotypal Traits; SOFAS, Social and Occupational Functioning Assessment Scale

**Table S8**. Predictors of studies with recovery data.

| **Author, year** | **Investigated predictors (N)** | **Predictors associated with recovery (N)** | **Predictor categories** | | | | | |
| --- | --- | --- | --- | --- | --- | --- | --- | --- |
|  |  |  | **Socio-demographic** | **Clinical** | **Pre-morbid** | **Cognitive** | **Neuro-imaging & biochemistry** | **Intervention** |
| Ajnakina, 2018[18] | Sexual abuse, physical abuse, parental separation, parental loss, institutional loss, institutional care, family arrangements, total adversity (7) | Total adversity more than 1 (1) |  | x |  |  |  |  |
| Austin, 2013[31] | Age, Working status, social contact weekly, GAF, baseline positive symptoms, negative symptoms, disorganized dimension, substance dependence, diagnosis (9) | Age, negative symptoms (2) | x | x |  |  |  |  |
| Álvarez-Jiménez, 2012[32] | Clinical, sociodemographic and psychosocial variables (38) | Sex, educational level, living status, premorbid adjustment, baseline negative symptoms (6) | x | x | x |  |  |  |
| Bjornestad, 2017[33] | Age, sex, substance abuse, DUP, baseline PANSS positive symptoms, negative symptoms, disorganized dimension, depressive dimension, Baseline social and family relationships items from Lehman’s Quality of Life Interview (9) | Substance abuse, frequency of social interactions with friends (2) | x | x |  |  |  |  |
| Chang, 2012[3] | Sex, age, educational level, employment status, age at onset, DUP, diagnosis, substance abuse, CGI-S positive and negative symptoms, symptom remission within first 3 months of treatment, number of hospitalisations, length of hospitalisations, suicide attempt, antipsychotic medication (15) | Educational level, DUP, vocational status (3) | x | x |  |  |  |  |
| Conus, 2017[6] | Age, sex, diagnosis, CGI severity of illness, CGI depression score, CGI mania score, employment status, education, insight, family history of psychotic disorders, premorbid functioning, traumatic events in the past, age at onset, DUP, duration of untreated prodrome, suicide attempt, forensic history, substance use disorder, past psychiatric disorder (19) | Duration of prodrome, CGI severity of illness, employment status, cannabis use at entry (4) | x | x |  |  |  |  |
| Evensen, 2012[55] | Apathy (1) | Apathy (1) |  | x |  |  |  |  |
| Faber, 2012[96] | Cannabis use (1) | Cannabis use (1) |  | x |  |  |  |  |
| Golay, 2019[9] | Migration (1) | Migration (1) | x |  |  |  |  |  |
| González-Blanch, 2010[97] | Age of onset, years of education, diagnosis, premorbid social adjustment, negative dimension, disorganized dimension, sustained attention (7) | Negative dimension, sustained attention (2) |  | x |  |  |  |  |
| Klaerke, 2019[34] | Age, sex, age at onset, cohort (4) | Sex (1) | x |  |  |  |  |  |
| Kurihara, 2011[102] | Age at onset, premorbid GAF, marital status, educational period, family history of psychosis, DUP (8) | DUP (1) |  | x |  |  |  |  |
| Lambert, 2006[65] | Age, sex, DUI, CGI severity, baseline PANSS positive symptoms, negative symptoms, general psychopathology, vocational status, independent living, subjective well-being, health related quality of life, treatment with antipsychotics before baseline, type of initial antipsychotic medication, side effects, substance use disorder, compliance (16) | Age, sex, employment status, independent living, treatment with antipsychotics before baseline, type of initial antipsychotic medication, early functional remission (7) | x | x |  |  |  |  |
| Lappin, 2018[35] | Clinic and sociodemographic variables | Mania and brief psychosis (1) | x |  |  |  |  |  |
| Manchanda, 2005[25] | Sex, marital status, history of drug abuse, EEG (4) | EEG, history of drug abuse (2) |  | x |  |  | x |  |
| O’Keeffe, 2019[16] | Premorbid adjustment, diagnosis, employment status, GAF, living alone, lifetime use of alcohol, DUP (7) | Employment status, premorbid adjustment, GAF, living alone (4) | x |  | x |  |  |  |
| Petersen, 2008[43] | Age, sex, DUP, academic, social, psychotic, and negative dimension, substance use, vocational education, adherence to medication, type of treatment (10) | Negative dimension, DUP, premorbid functioning, substance abuse, adherence to medication, type of treatment: OPUS vs standard treatment (6) |  | x | x |  |  | x |
| Phahladira, 2020[37] | PANSS positive symptoms, negative symptoms, general psychopathology, SOFAS, premorbid adjustment, substance use, ethnicity, quality of life, DUP (9) | Substance use, ethnicity, premorbid functioning (3) | x | x | x |  |  |  |
| Robinson, 2004[22] | Age, sex, ethnicity, education, social class, duration of psychosis symptoms at entry, diagnosis, positive symptoms, negative symptoms, premorbid social adjustment, cognitive domains: language, memory, attention, executive function, motor function, visuospatial function, premorbid cognitive functioning, social adjustment, MRI brain volumes, time taking antipsychotic medication (20) | Cognition (global), composite index of cortical asymmetry (2) |  |  |  | x | x |  |
| Simonsen, 2017[78] | DUP, PANSS positive symptoms, negative symptoms, disorganized symptoms, GAF, social functioning during childhood, neurocognitive domains: current IQ, verbal learning and memory, processing speed, working memory, verbal fluency, interference control (12) | Not significant |  |  |  |  |  |  |
| Shrivastava, 2010[38] | Age at onset, baseline PANSS positive symptoms, negative symptoms, general psychopathology, HDRS score, employment status, independent living, symptoms of aggression (11) | Age at onset, baseline PANSS positive symptoms, negative symptoms, general psychopathology, HDRS score, employment status, independent living, symptoms of aggression (8) | x | x |  |  |  |  |
| Sullivan, 2018[106] | DUP, positive symptoms, negative symptoms (3) | DUP, positive symptoms (2) |  | x |  |  |  |  |
| Thorup, 2014[39] | Sex (1) | Sex (1) | x |  |  |  |  |  |
| Torgalsbøen, 2015[21] | Years of education, PANSS positive symptoms, attention/vigilance, reasoning/problem solving (4) | Years of education, attention/vigilance (2) | x |  |  | x |  |  |
| Treen Calvo,2018[42] | Age, sex, education, family history of psychosis, hospitalization at intake, antipsychotic medication, DUP, positive and negative symptoms, Calgary depression scale, insight, premorbid adjustment during childhood, premorbid adjustment during adolescence, premorbid adjustment in adulthood, premorbid IQ, verbal memory, visual memory, executive functions, working memory, speed of processing, attention (21) | Premorbid adjustment during childhood, premorbid adjustment during adolescence, speed of processing, executive function (4) |  |  | x | x |  |  |
| Verma, 2012[14] | Age, sex, marital status, education, ethnicity, diagnosis, early improvement at month 3, DUP, baseline PANSS positive symptoms, negative symptoms, general psychopathology, GAF (12) | Age, sex, marital status, education, ethnicity, early improvement at month 3, DUP, baseline PANSS negative symptoms (8) | x | x |  |  |  |  |
| Wunderink, 2008[88] | Discontinuation of antipsychotics versus maintenance treatment (1) | Not significant |  |  |  |  |  |  |
| Wunderink, 2013[40] | DUP, social functioning, living alone, treatment: dose reduction/discontinuation of antipsychotics (DR) versus maintenance treatment (MT) (4) | Social functioning, treatment: dose reduction/discontinuation of antipsychotics (DR) versus maintenance treatment (MT) (2) | x | x |  |  |  | x |

BMI, body mass index; BPRS, Brief Psychiatric Rating Scale; CGI, Clinical Global Impression; DUI, duration of untreated illness; DUP, duration of untreated psychosis; GAF, Global Assessment of Functioning; PANSS, Positive and Negative Symptom Scale; PAS, Premorbid Adjustment Scale; PSST, Premorbid Schizoid and Schizotypal Traits; SOFAS, Social and Occupational Functioning Assessment Scale

**Figure S1**. Forest plot remission data

**Figure S2.** Forest plot recovery data

**Figure S3**. Funnel plot for remission studies

**Figure S4.** Funnel plot for recovery studies

References

[1] Andreasen NC, Carpenter WT, Kane JM, Lasser RA, Marder SR, Weinberger DR. Remission in schizophrenia: Proposed criteria and rationale for consensus. American Journal of Psychiatry. 2005;162:441-9.

[2] Egger M, Davey Smith G, Schneider M, Minder C. Bias in meta-analysis detected by a simple, graphical test. BMJ (Clinical research ed). 1997;315:¡629-34.

[3] Chang WC, Tang JY, Hui CL, Lam MM, Chan SK, Wong GH, et al. Prediction of remission and recovery in young people presenting with first-episode psychosis in Hong Kong: A 3-year follow-up study. Australian and New Zealand Journal of Psychiatry. 2012;46:100-8.

[4] Chang WC, Chan TC, Chen ES, Hui CL, Wong GH, Chan SK, et al. The concurrent and predictive validity of symptomatic remission criteria in first-episode schizophrenia. Schizophr Res. 2013;143:107-15.

[5] Colizzi M, Carra E, Fraietta S, Lally J, Quattrone D, Bonaccorso S, et al. Substance use, medication adherence and outcome one year following a first episode of psychosis. Schizophr Res. 2016;170:311-7.

[6] Conus P, Cotton SM, Francey SM, O'Donoghue B, Schimmelmann BG, McGorry PD, et al. Predictors of favourable outcome in young people with a first episode psychosis without antipsychotic medication. Schizophrenia Research. 2017;185:130-6.

[7] Dazzan P, Lappin JM, Heslin M, Donoghue K, Lomas B, Reininghaus U, et al. Symptom remission at 12-weeks strongly predicts long-term recovery from the first episode of psychosis. Psychological medicine. 2019:1-11.

[8] Gaebel W, Riesbeck M, Woelwer W, Klimke A, Eickhoff M, von Wilmsdorff M, et al. Rates and predictors of remission in first-episode schizophrenia within 1 year of antipsychotic maintenance treatment. Results of a randomized controlled trial within the German Research Network on Schizophrenia. Schizophrenia Research. 2014;152:478-86.

[9] Golay P, Baumann PS, Jaton L, Restellini R, Solida A, Mebdouhi N, et al. Migration in patients with early psychosis: A 3-year prospective follow-up study. Psychiatry Research. 2019;275:108-14.

[10] Kaminga AC, Dai W, Liu A, Myaba J, Banda R, Wen SW, et al. Rate of and time to symptomatic remission in first-episode psychosis in Northern Malawi: A STROBE-compliant article. Medicine (Baltimore). 2018;97:e13078.

[11] Lambert M, Schimmelmann BG, Naber D, Schacht A, Karow A, Wagner T, et al. Prediction of remission as a combination of symptomatic and functional remission and adequate subjective well-being in 2960 patients with schizophrenia. Journal of Clinical Psychiatry. 2006;67:1690-7.

[12] Renwick L, Lyne J, Donoghue BO, Owens L, Doyle R, Hill M, et al. Prodromal symptoms and remission following first episode psychosis. Schizophr Res. 2015;168:30-6.

[13] Ucok A, Serbest S, Kandemir PE. Remission after first-episode schizophrenia: results of a long-term follow-up. Psychiatry Res. 2011;189:33-7.

[14] Verma S, Subramaniam M, Abdin E, Poon LY, Chong SA. Symptomatic and functional remission in patients with first-episode psychosis. Acta Psychiatrica Scandinavica. 2012;126:282-9.

[15] Addington J, Addington D. Symptom remission in first episode patients. Schizophrenia Research. 2008;106:281-5.

[16] O'Keeffe D, Hannigan A, Doyle R, Kinsella A, Sheridan A, Kelly A, et al. The iHOPE-20 study: Relationships between and prospective predictors of remission, clinical recovery, personal recovery and resilience 20 years on from a first episode psychosis. Aust N Z J Psychiatry. 2019;53:1080-92.

[17] Tang SX, Yi JJ, Moore TM, Calkins ME, Kohler CG, Whinna DA, et al. Subthreshold Psychotic Symptoms in 22q11.2 Deletion Syndrome. Journal of the American Academy of Child and Adolescent Psychiatry. 2014;53:991-1000.

[18] Ajnakina O, Trotta A, Di Forti M, Stilo SA, Kolliakou A, Gardner-Sood P, et al. Different types of childhood adversity and 5-year outcomes in a longitudinal cohort of first-episode psychosis patients. Psychiatry Research. 2018;269:199-206.

[19] Boden R, Abrahamsson T, Holm G, Borg J. Psychomotor and cognitive deficits as predictors of 5-year outcome in first-episode schizophrenia. Nord J Psychiatry. 2014;68:282-8.

[20] Benoit A, Bodnar M, Malla AK, Joober R, Bherer L, Lepage M. Changes in memory performance over a 12-month period in relation to achieving symptomatic remission after a first-episode psychosis. Schizophr Res. 2014;153:103-8.

[21] Torgalsboen AK, Mohn C, Czajkowski N, Rund BR. Relationship between neurocognition and functional recovery in first-episode schizophrenia: Results from the second year of the Oslo multi-follow-up study. Psychiatry Res. 2015;227:185-91.

[22] Robinson DG, Woerner MG, McMeniman M, Mendelowitz A, Bilder RM. Symptomatic and functional recovery from a first episode of schizophrenia or schizoaffective disorder. Am J Psychiatry. 2004;161:473-9.

[23] Jauhar S, Veronese M, Nour MM, Rogdaki M, Hathway P, Turkheimer FE, et al. Determinants of treatment response in first-episode psychosis: an F-18-DOPA PET study. Molecular Psychiatry. 2019;24:1502-12.

[24] Yee JY, Lee T-S, Lee J. A Longitudinal Study of Serum Brain-Derived Neurotrophic Factor Levels in First-Episode Schizophrenia. Journal of Clinical Psychopharmacology. 2019;39:639-43.

[25] Manchanda R, Norman RM, Malla AK, Harricharan R, Northcott S. Persistent psychoses in first episode patients. Schizophr Res. 2005;80:113-6.

[26] Green AI, Lieberman JA, Hamer RM, Glick ID, Gur RE, Kahn RS, et al. Olanzapine and haloperidol in first episode psychosis: Two-year data. Schizophrenia Research. 2006;86:234-43.

[27] Boter H, Peuskens J, Libiger J, Fleischhacker WW, Davidson M, Galderisi S, et al. Effectiveness of antipsychotics in first-episode schizophrenia and schizophreniform disorder on response and remission: An open randomized clinical trial (EUFEST). Schizophrenia Research. 2009;115:97-103.

[28] Barrio P, Batalla A, Castellvi P, Hidalgo D, Garcia M, Ortiz A, et al. Effectiveness of long-acting injectable risperidone versus oral antipsychotics in the treatment of recent-onset schizophrenia: a case-control study. International Clinical Psychopharmacology. 2013;28:164-70.

[29] Malla A, Joober R, Iyer S, Norman R, Schmitz N, Brown T, et al. Comparing three-year extension of early intervention service to regular care following two years of early intervention service in first-episode psychosis: a randomized single blind clinical trial. World Psychiatry. 2017;16:278-86.

[30] Tempier R, Balbuena L, Lepnurm M, Craig TKJ. Perceived emotional support in remission: results from an 18-month follow-up of patients with early episode psychosis. Social Psychiatry and Psychiatric Epidemiology. 2013;48:1897-904.

[31] Austin SF, Mors O, Secher RG, Hjorthoj CR, Albert N, Bertelsen M, et al. Predictors of recovery in first episode psychosis: The OPUS cohort at 10 year follow-up. Schizophrenia Research. 2013;150:163-8.

[32] Alvarez-Jimenez M, Parker AG, Hetrick SE, McGorry PD, Gleeson JF. Preventing the Second Episode: A Systematic Review and Meta-analysis of Psychosocial and Pharmacological Trials in First-Episode psychosis. Schizophrenia Bulletin. 2011;37:619-30.

[33] Bjornestad J, Hegelstad WtV, Joa I, Davidson L, Larsen TK, Melle I, et al. "With a little help from my friends" social predictors of clinical recovery in first-episode psychosis. Psychiatry Research. 2017;255:209-14.

[34] Klaerke LR, Baandrup L, Fagerlund B, Ebdrup BH, Pantelis C, Glenthoj BY, et al. Diagnostic stability and long-term symptomatic and functional outcomes in first-episode antipsychotic-naive patients with schizophrenia. European Psychiatry. 2019;62:130-7.

[35] Lappin JM, Heslin M, Lomas B, Jones PB, Doody GA, Reininghaus UA, et al. Early sustained recovery following first episode psychosis: Evidence from the AESOP10 follow-up study. Schizophr Res. 2018;199:341-5.

[36] O'Keeffe D, Hannigan A, Doyle R, Kinsella A, Sheridan A, Kelly A, et al. 20 Year Outcome in Psychotic Illness: Initial Findings of the iHOPE-20 First Episode Psychosis Incidence Cohort Follow up Study. Early Intervention in Psychiatry. 2018;12:60-.

[37] Phahladira L, Luckhoff HK, Asmal L, Kilian S, Scheffler F, Plessis SD, et al. Early recovery in the first 24 months of treatment in first-episode schizophrenia-spectrum disorders. NPJ Schizophr. 2020;6:2.

[38] Shrivastava A, Shah N, Johnston M, Stitt L, Thakar M. Predictors of long-term outcome of first-episode schizophrenia: A ten-year follow-up study. Indian J Psychiatry. 2010;52:320-6.

[39] Thorup A, Albert N, Bertelsen M, Petersen L, Jeppesen P, Le Quack P, et al. Gender differences in first-episode psychosis at 5-year follow-up - two different courses of disease? Results from the OPUS study at 5-year follow-up. European Psychiatry. 2014;29:44-51.

[40] Wunderink L, Nieboer RM, Wiersma D, Sytema S, Nienhuis FJ. Recovery in Remitted First-Episode Psychosis at 7 Years of Follow-up of an Early Dose Reduction/Discontinuation or Maintenance Treatment Strategy Long-term Follow-up of a 2-Year Randomized Clinical Trial. Jama Psychiatry. 2013;70:913-20.

[41] Petersen TJ, Huffman JC, Weiss AP, Blais MA, Andreotti CF, Horwitz MB, et al. Reach of benchmark psychiatric trial results to community-based providers: A case study of CATIE. Journal of Clinical Psychiatry. 2008;69:1081-6.

[42] Treen Calvo D, Gimenez-Donoso S, Setien-Suero E, Toll Privat A, Crespo-Facorro B, Ayesa Arriola R. Targeting recovery in first episode psychosis: The importance of neurocognition and premorbid adjustment in a 3-year longitudinal study. Schizophrenia Research. 2018;195:320-6.

[43] Petersen L, Thorup A, Oqhlenschlaeger J, Christensen TO, Jeppesen P, Krarup G, et al. Predictors of Remission and Recovery in a First-Episode Schizophrenia Spectrum Disorder Sample: 2-Year Follow-Up of the OPUS Trial. Canadian Journal of Psychiatry-Revue Canadienne De Psychiatrie. 2008;53:660-70.

[44] Alaghband-rad J, Boroumand M, Amini H, Sharifi V, Omid A, Davari-Ashtiani R, et al. Non-affective Acute Remitting Psychosis: a preliminary report from Iran. Acta Psychiatr Scand 2006;113:96-101.

[45] Ceskova E, Prikryl R, Kasparek T, Kuccerova H. Insight in first-episode schizophrenia. Int J Psychiatry Clin Pract. 2008;12:36-40.

[46] Ceskova E, Prikryl R, Kasparek T. Outcome in males with first-episode schizophrenia: 7-year follow-up. World J Biol Psychiatry. 2011;12:66-72.

[47] Chang WC, Tang JY, Hui CL, Lam MM, Chan SK, Wong GH, et al. Prediction of remission and recover in young people presenting with first-episode psychosis in Hong Kong: A 3-year follow-up study. Australian & New Zealand Journal of Psychiatry

. 2012;46:100–8.

[48] Clausen L, Hjorthoj CR, Thorup A, Jeppesen P, Petersen L, Bertelsen M, et al. Change in cannabis use, clinical symptoms and social functioning among patients with first-episode psychosis: a 5-year follow-up study of patients in the OPUS trial. Psychological Medicine. 2014;44:117-26.

[49] Crespo-Facorro B, Perez-Iglesias R, Mata I, Caseiro O, Martinez-Garcia O, Pardo G, et al. Relapse prevention and remission attainment in first-episode non-affective psychosis. A randomized, controlled 1-year follow-up comparison of haloperidol, risperidone and olanzapine. Journal of Psychiatric Research. 2011;45:763-9.

[50] Crumlish N, Whitty P, Clarke M, Browne S, Kamali M, Gervin M, et al. Beyond the critical period: longitudinal study of 8-year outcome in first-episode non-affective psychosis. British Journal of Psychiatry. 2009;194:18-24.

[51] de Haan L, Nimwegen L, Amelsvoort T, Dingemans P, Linszen D. Improvement of subjective well-being and enduring symptomatic remission, a 5-year follow-up of first episode schizophrenia. Pharmacopsychiatry. 2008;41:125-8.

[52] DeLisi LE, Sakuma M, Ge S, Kushner M. Association of brain structural change with the heterogeneous course of schizophrenia from early childhood through five years subsequent to a first hospitalization. Psychiatry Res. 1998;84:75-88.

[53] Drake RJ, Day CJ, Picucci R, Warburton J, Larkin W, Husain N, et al. A naturalistic, randomized, controlled trial combining cognitive remediation with cognitive-behavioural therapy after first-episode non-affective psychosis. Psychological Medicine. 2014;44:1889-99.

[54] Emsley R, Rabinowitz J, Medori R, Early Psychosis Global Working G. Remission in early psychosis: Rates, predictors, and clinical and functional outcome correlates. Schizophrenia Research. 2007;89:129-39.

[55] Evensen J, Rossberg JI, Barder H, Haahr U, Hegelstad WtV, Joa I, et al. Apathy in first episode psychosis patients: A ten year longitudinal follow-up study. Schizophrenia Research. 2012;136:19-24.

[56] Fraguas D, del Rey-Mejias A, Moreno C, Castro-Fornieles J, Graell M, Otero S, et al. Duration of untreated psychosis predicts functional and clinical outcome in children and adolescents with first-episode psychosis: A 2-year longitudinal study. Schizophrenia Research. 2014;152:130-8.

[57] Gaebel W, Riesbeck M, Woelwer W, Klimke A, Eickhoff M, von Wilmsdorff M, et al. Maintenance treatment with risperidone or low-dose haloperidol in first-episode schizophrenia: 1-Year results of a randomized controlled trial within the German research network on schizophrenia. Journal of Clinical Psychiatry. 2007;68:1763-74.

[58] Gasquet I, Haro JM, Tcherny-Lessenot S, Chartier F, Lepine J-P. Remission in the outpatient care of schizophrenia: 3-Year results from the Schizophrenia Outpatients Health Outcomes (SOHO) Study in France. European Psychiatry. 2008;23:491-6.

[59] Hassan GA, Taha GR. Long term functioning in early onset psychosis: two years prospective follow-up study. Behav Brain Funct. 2011;7:28.

[60] Hill M, Crumlish N, Clarke M, Whitty P, Owens E, Renwick L, et al. Prospective relationship of duration of untreated psychosis to psychopathology and functional outcome over 12 years. Schizophrenia Research. 2012;141:215-21.

[61] Ho BC, Andreasen NC, Flaum M, Nopoulos P, Miller D. Untreated initial psychosis: its relation to quality of life and symptom remission in first-episode schizophrenia. Am J Psychiatry. 2000;157:808-15.

[62] Jablensky A, Sartorius N, Ernberg G, Anker M, Korten A, Cooper JE, et al. Schizophrenia: manifestations, incidence and course in different cultures. A World Health Organization ten-country study. Psychol Med Monogr Suppl. 1992;20:1-97.

[63] Johnson S, Sathyaseelan M, Charles H, Jacob KS. Predictors of disability: a 5-year cohort study of first-episode schizophrenia. Asian journal of psychiatry. 2014;9:45-50.

[64] Kinoshita A, Nakane Y, Nakane H, Ishizaki Y, Honda S, Ohta Y, et al. Nagasaki Schizophrenia Study: Influence of the Duration of Untreated Psychosis on Long-Term Outcome. Acta Med Nagasaki 2005;50:17-22.

[65] Lambert M, Schimmelmann BG, Naber D, Schacht A, Karow A, Wagner T, et al. Prediction of remission as a combination of symptomatic and functional remission and adequate subjective well-being in 2960 patients with schizophrenia. J Clin Psychiatry. 2006;67:1690-7.

[66] Langeveld J, Joa I, Friis S, Hegelstad WtV, Melle I, Johannessen JO, et al. A comparison of adolescent- and adult-onset first-episode, non-affective psychosis: 2-year follow-up. European Archives of Psychiatry and Clinical Neuroscience. 2012;262:599-605.

[67] Lieberman JA, Alvir JM, Woerner M, Degreef G, Bilder RM, Ashtari M, et al. Prospective study of psychobiology in first-episode schizophrenia at Hillside Hospital. Schizophr Bull. 1992;18:351-71.

[68] Spitzer RL, Endicott J. Schedule for Affective Disorders and Schizophrenia—Change Version. 3rd ed New York: New York State Psychiatric Institute; 1978.

[69] Luckhoff H, Phahladira L, Scheffler F, Asmal L, du Plessis S, Chiliza B, et al. Weight gain and metabolic change as predictors of symptom improvement in first-episode schizophrenia spectrum disorder patients treated over 12 months. Schizophrenia Research. 2019;206:171-6.

[70] Mojtabai R, Lavelle J, Gibson PJ, Bromet EJ. Atypical antipsychotics in first admission schizophrenia: Medication continuation and outcomes. Schizophrenia Bulletin. 2003;29:519-30.

[71] Mustafa S, Joober R, Lepage M, Iyer S, Shah J, Malla A. Predictors of 'all-cause discontinuation' of initial oral antipsychotic medication in first episode psychosis. Schizophr Res. 2018;201:287-93.

[72] Nishida A, Ando S, Yamasaki S, Koike S, Ichihashi K, Miyakoshi Y, et al. A randomized controlled trial of comprehensive early intervention care in patients with first-episode psychosis in Japan: 1.5-year outcomes from the J-CAP study. Journal of Psychiatric Research. 2018;102:136-41.

[73] Norman RM, Manchanda R, Windell D. The prognostic significance of early remission of positive symptoms in first treated psychosis. Psychiatry Res. 2014;218:44-7.

[74] Perkins DO, Lieberman JA, Gu HB, Tohen M, McEvoy J, Green AI, et al. Predictors of antipsychotic treatment response in patients with first-episode schizophrenia, schizoaffective and schizophreniform disorders. British Journal of Psychiatry. 2004;185:18-24.

[75] Saravanan B, Jacob KS, Johnson S, Prince M, Bhugra D, David AS. Outcome of first-episode schizophrenia in India: longitudinal study of effect of insight and psychopathology. Br J Psychiatry. 2010;196:454-9.

[76] Schennach-Wolff R, Jaeger M, Mayr A, Meyer S, Kuehn K-U, Klingberg S, et al. Predictors of response and remission in the acute treatment of first-episode schizophrenia patients - Is it all about early response? European Neuropsychopharmacology. 2011;21:370-8.

[77] Schennach R, Riesbeck M, Mayr A, Seemueller F, Maier W, Klingberg S, et al. Should early improvement be re-defined to better predict the maintenance of response in first-episode schizophrenia patients? Acta Psychiatrica Scandinavica. 2013;127:474-81.

[78] Simonsen C, Faerden A, Romm KL, Berg AO, Bjella T, Sundet K, et al. Early clinical recovery in first-episode psychosis: Symptomatic remission and its correlates at 1-year follow-up. Psychiatry Res. 2017;254:118-25.

[79] Simonsen E, Friis S, Opjordsmoen S, Mortensen EL, Haahr U, Melle I, et al. Early identification of non-remission in first-episode psychosis in a two-year outcome study. Acta Psychiatr Scand. 2010;122:375-83.

[80] Stralin P, Skott M, Cullberg J. Early recovery and employment outcome 13 years after first episode psychosis. Psychiatry Research. 2019;271:374-80.

[81] Takeuchi H, Siu C, Remington G, Fervaha G, Zipursky RB, Foussias G, et al. Does relapse contribute to treatment resistance? Antipsychotic response in first- vs. second-episode schizophrenia. Neuropsychopharmacology. 2019;44:1036-42.

[82] Tang JY-M, Chang W-C, Hui CL-M, Wong GH-Y, Chan SK-W, Lee EH-M, et al. Prospective relationship between duration of untreated psychosis and 13-year clinical outcome: A first-episode psychosis study. Schizophrenia Research. 2014;153:1-8.

[83] Thara R. Twenty-Year Course of Schizophrenia: The Madras Longitudinal Study. Canadian Journal of Psychiatry-Revue Canadienne De Psychiatrie. 2004;49:564-9.

[84] Torgalsboen A-K, Mohn C, Rund BR. Neurocognitive predictors of remission of symptoms and social and role functioning in the early course of first-episode schizophrenia. Psychiatry Research. 2014;216:1-5.

[85] Vazquez-Barquero JL, Cuesta MJ, Castanedo SH, Lastra I, Herran A, Dunn G. Cantabria first-episode schizophrenia study: three-year follow-up. British Journal of Psychiatry. 1999;174:141-9.

[86] Wade D, Harrigan S, Edwards J, Burgess PM, Whelan G, McGorry PD. Substance misuse in first-episode psychosis: 15-month prospective follow-up study. Br J Psychiatry. 2006;189:229-34.

[87] Wunderink L, Nienhuis FJ, Sytema S, Wiersma D. Predictive validity of proposed remission criteria in first-episode schizophrenic patients responding to antipsychotics. Schizophr Bull. 2007;33:792-6.

[88] Wunderink L, Nienhuis F, Sytema S, Wiersma D. Guided discontinuation trial versus maintenance treatment in remitted first episode psychosis: relapse rates and functional outcome; the MESIFOS RCT. Early Intervention in Psychiatry. 2008;2:A57-A.

[89] Xenaki L-A, Kollias CT, Stefanatou P, Ralli I, Soldatos R-F, Dimitrakopoulos S, et al. Organization framework and preliminary findings from the Athens First-Episode Psychosis Research Study. Early Intervention in Psychiatry. 2019.

[90] Zarate CA, Jr., Tohen M, Land ML. First-episode schizophreniform disorder: comparisons with first-episode schizophrenia. Schizophr Res. 2000;46:31-4.

[91] Zhang C, Chen M-J, Wu G-J, Wang Z-W, Rao S-Z, Zhang Y, et al. Effectiveness of Antipsychotic Drugs for 24-Month Maintenance Treatment in First-Episode Schizophrenia: Evidence From a Community-Based "Real-World" Study. Journal of Clinical Psychiatry. 2016;77:E1460-E6.

[92] Zhou F-C, Wang C-Y, Ungvari GS, Ng CH, Zhou Y, Zhang L, et al. Longitudinal changes in prospective memory and their clinical correlates at 1-year follow-up in first-episode schizophrenia. Plos One. 2017;12.

[93] Bachmann S, Bottmer C, Schroder J. One-year outcome and its prediction in first-episode schizophrenia--a naturalistic study. Psychopathology. 2008;41:115-23.

[94] Bertelsen M, Jeppesen P, Petersen L, Thorup A, Ohlenschlaeger J, Le Quach P, et al. Course of illness in a sample of 265 patients with first-episode psychosis--five-year follow-up of the Danish OPUS trial. Schizophr Res. 2009;107:173-8.

[95] Craig TJ, Bromet EJ, Fennig S, Tanenberg-Karant M, Lavelle J, Galambos N. Is there an association between duration of untreated psychosis and 24-month clinical outcome in a first-admission series? American Journal of Psychiatry. 2000;157:60-6.

[96] Faber G, Smid HGOM, Van Gool AR, Wunderink L, van den Bosch RJ, Wiersma D. Continued Cannabis Use and Outcome in First-Episode Psychosis: Data From a Randomized, Open-Label, Controlled Trial. Journal of Clinical Psychiatry. 2012;73:632-8.

[97] Gonzalez-Blanch C, Perez-Iglesias R, Pardo-Garcia G, Rodriguez-Sanchez JM, Martinez-Garcia O, Vazquez-Barquero JL, et al. Prognostic value of cognitive functioning for global functional recovery in first-episode schizophrenia. Psychological Medicine. 2010;40:935-44.

[98] Harrison G, Hopper K, Craig T, Laska E, Siegel C, Wanderling J, et al. Recovery from psychotic illness: a 15-and 25-year international follow-up study. British Journal of Psychiatry. 2001;178:506-17.

[99] Harrow M, Sands JR, Silverstein ML, Goldber JF. Course and Outcome for Schizophrenia Versus Other Psychotic Patients: A Longitudinal Study. Schizophr Bull. 1997;23:287-303.

[100] Harrow M, Grossman LS, Jobe TH, Herbener ES. Do patients with schizophrenia ever show periods of recovery? A 15-year multi-follow-up study. Schizophrenia Bulletin. 2005;31:723-34.

[101] Lieberman RP, Kopelowicz A, Ventura J, Gutkind D. Operational criteria and factors related to recovery from schizophrenia. Int Rev Psychiatry. 2002;14:256–72.

[102] Kurihara T, Kato M, Reverger R, Tirta IG. Seventeen-year clinical outcome of schizophrenia in Bali. Eur Psychiatry. 2011;26:333-8.

[103] Mattsson M, Topor A, Cullberg J, Forsell Y. Association between financial strain, social network and five-year recovery from first episode psychosis. Soc Psychiatry Psychiatr Epidemiol. 2008;43:947-52.

[104] Rangaswamy T, Mangala R, Mohan G, Joseph J, John S. Intervention for first episode psychosis in India - The SCARF experience. Asian J Psychiatr. 2012;5:58-62.

[105] Rangaswamy T. Twenty-five years of schizophrenia: The Madras longitudinal study. Indian J Psychiatry. 2012;54:134-7.

[106] Sullivan SA, Carroll R, Peters TJ, Amos T, Jones PB, Marshall M, et al. Duration of untreated psychosis and clinical outcomes of first episode psychosis: An observational and an instrumental variables analysis. Early Intervention in Psychiatry. 2019;13:841-7.

[107] Bebbington PE, Angermeyer M, Azorin JM, Brugha T, Kilian R, Johnson S, et al. The European Schizophrenia Cohort (EuroSC) - A naturalistic prognostic and economic study. Social Psychiatry and Psychiatric Epidemiology. 2005;40:707-17.

[108] Tarricone I, Boydell J, Panigada S, Allegri F, Marcacci T, Minenna MG, et al. The impact of substance use at psychosis onset on First Episode Psychosis course: Results from a 1 year follow-up study in Bologna. Schizophrenia Research. 2014;153:60-3.

[109] Selten J-P, Veen ND, Hoek HW, Laan W, Schols D, van der Tweel I, et al. Early course of schizophrenia in a representative Dutch incidence cohort. Schizophrenia Research. 2007;97:79-87.

[110] Valencia M, Juarez F, Ortega H. Integrated treatment to achieve functional recovery for first-episode psychosis. Schizophr Res Treatment. 2012;2012:962371.

[111] Torgalsboen AK. Full recovery from schizophrenia: the prognostic role of premorbid adjustment, symptoms at first admission, precipitating events and gender. Psychiatry Res. 1999;88:143-52.

[112] VanOs J, Fahy TA, Jones P, Harvey I, Sham P, Lewis S, et al. Psychopathological syndromes in the functional psychoses: Associations with course and outcome. Psychological Medicine. 1996;26:161-76.
